# Supplementary material for: LARGE1 processively polymerizes length-controlled matriglycan on prodystroglycan
Source: Nat Commun. 2025 Oct 10;16:9028. doi: 10.1038/s41467-025-64080-z (PMC12514197; doi:10.1038/s41467-025-64080-z)

Supplementary Information for

LARGE1 processively polymerizes length-controlled matriglycan on prodystroglycan

Soumya Joseph, Nicholas J Schnicker, Nicholas Spellmon, Zhen Xu, Rui Yan, Zhiheng Yu,

Omar Davulcu, Tiandi Yang, Jesse Hopkins, Mary E Anderson, David Venzke,

Kevin P Campbell

This file contains:
Supplementary Figures 1-18
Supplementary Tables 1-5
Gel Source Data for: S12, S13, S15a-c

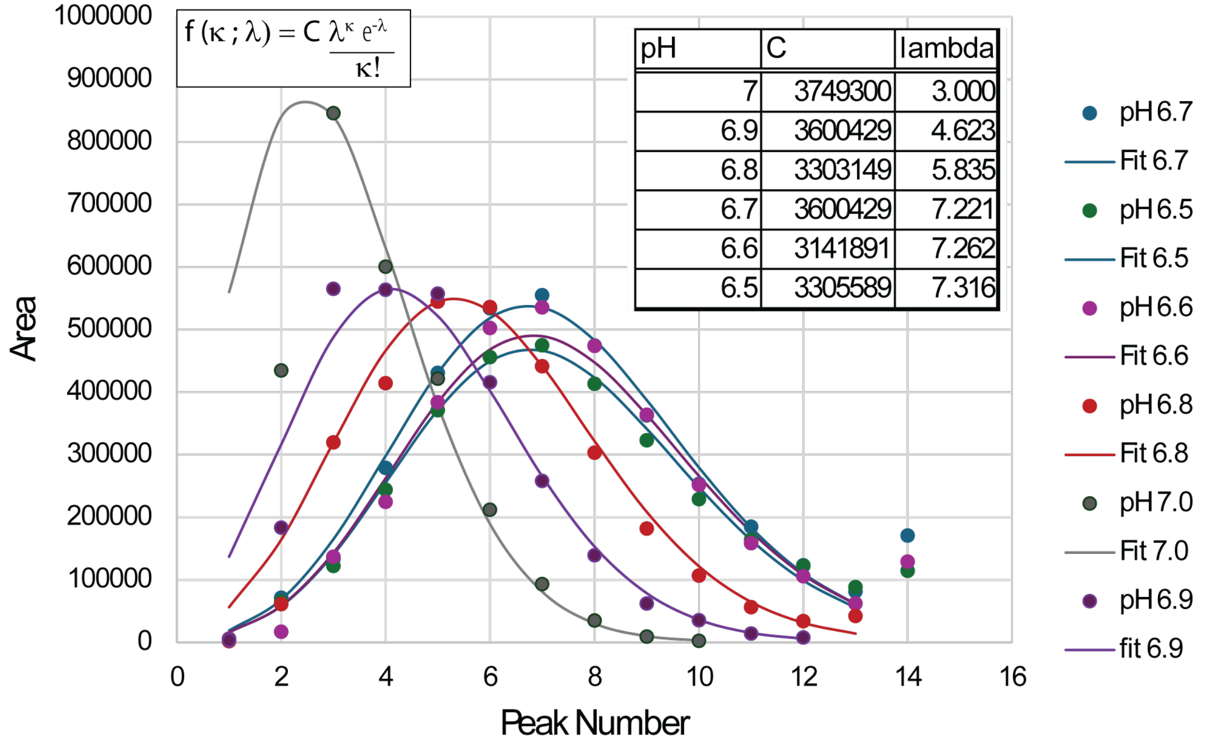

Supplementary Fig. 1. Poisson distribution of product based on length (peak number). The area under the curve of each peak (y-axis) from Fig. 1a is plotted against peak number (x-axis). Insets show the Poisson equation (left panel) and a table with values obtained by minimizing the absolute difference between observed and theoretical distributions (right panel). Peaks at higher retention times (< 40 minutes) could not be resolved and were integrated as a single peak, which explains the increased area of the last data point at lower pH values (6.5-6.7). Higher pH (6.9-7.0) product distributions do not fit the Poisson distribution because LARGE1 does not efficiently polymerize matriglycan at neutral pH. Source data are provided as a Source Data file.

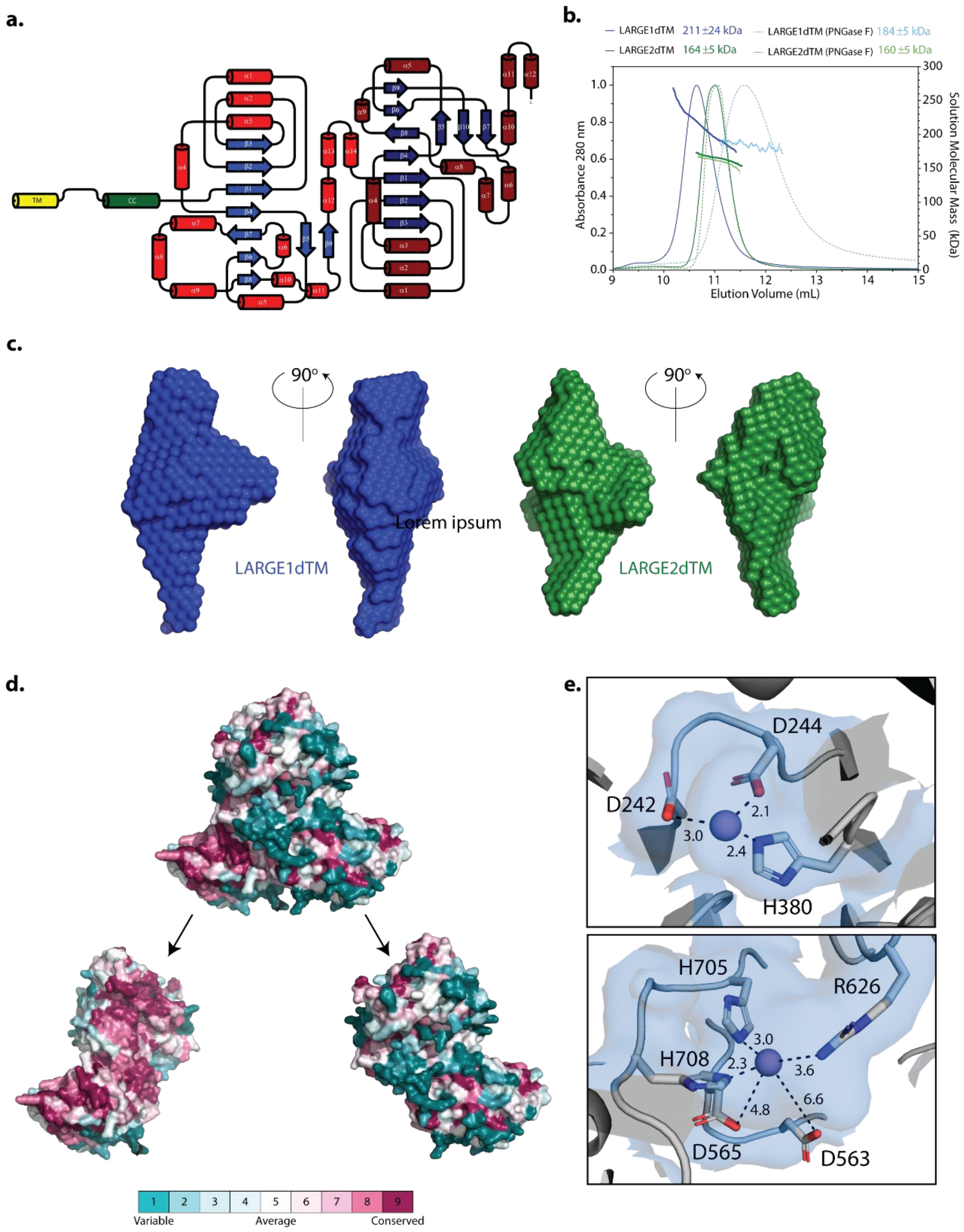

Supplementary Fig. 2. LARGE1 structure and active sites. a. Schematic showing the secondary structure of a LARGE1 protomer. Alpha-helices are represented by cylinders, and beta-strands are represented by arrows. LARGE1 consists of a single-pass transmembrane (TM, yellow) domain and a coiled-coil domain (CC, green), followed by tandem Rossmann-like folds with a core of at least seven-stranded beta-sheet (blue) surrounded by alpha-helices (red/maroon). The catalytic, manganese-chelating, DXD motif is located between beta-strands four and five in each domain. **b.** Size-exclusion chromatography and in-line multi-angle light scattering (SEC-MALS) show

LARGEdTM form dimers in solution. The elution profile (continuous line) was monitored by a change in refractive index and absorbance at 280 nm (primary y-axis), whereas discrete points show the weight-average molecular weights across peaks (secondary y-axis). **c.** Small-angle X-ray scattering (SEC-MALS-SAXS) was used to calculate molecular envelopes for LARGEdTM constructs. **d.** Surface conservation of catalytic domains (ConSurf server: https://consurf.tau.ac.il/consurf_index.php with default settings) of LARGE1 dimer (top panel) and individual protomers (bottom panel). **e.** Residues in the glucuronic acid transferase domain (chain A) that are possibly involved in Mn^{2+} coordination. Map density is shown as a blue surface at 1 sigma. Distances (Å) are shown both for coordinating and possible coordinating residues. Source data are provided as a Source Data file.

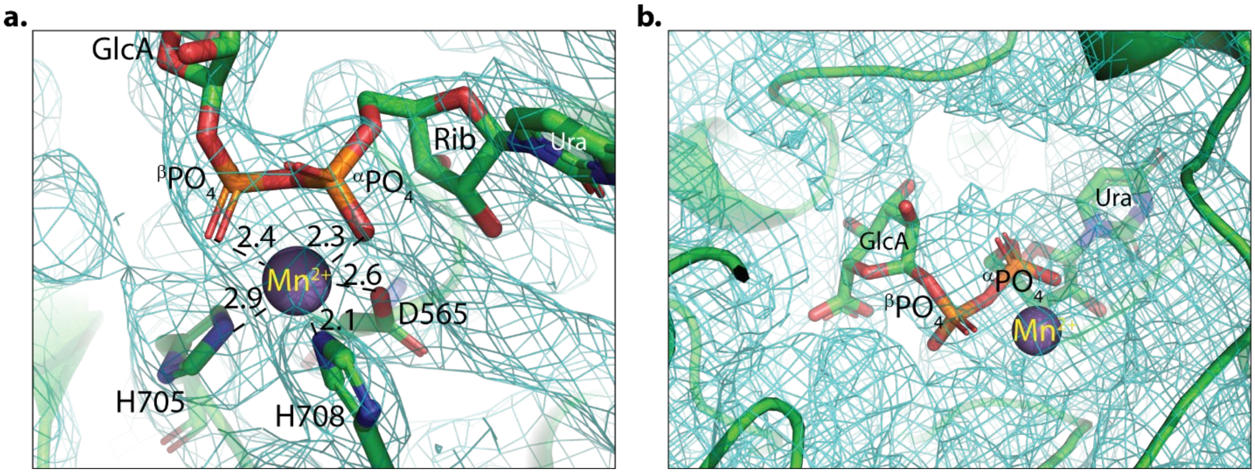

Supplementary Fig. 3. Reconstructed density in active sites of LARGE1dTM. a. Reconstructed volume in UDP-glucuronate transferase active site. The coordinating residues (D565, H705 and H708), phosphate moieties (PO_4), uridine (Ura) and glucuronate (GlcA) from UDP-glucuronate are labelled. Clear spherical density was not discernible for manganese ions (Mn^{2+}) in active sites but were inferred from density contiguous with coordinating residues as well as the diphosphate from UDP-glucuronate, and their bond distances (Å). **b.** Another view of the active site to include density for glucuronate (GlcA) unobstructed by other densities.

Transmembrane Coiled-coil Mn binding DXD Mn binding C-His

```
LARGE1 MLGICRGRRKFLAASLSLLCIPAITWIYLFSGSFEDGKPVSLSPLESQAHSPRYTASSQR 60
LARGE2 --MLPRGRPRALGAAAALLLLLLL-GFLLFGGDLGCERREP----GGRAGAPGCFPGP-- 51
         : *** : *.*:  ** :  :  : **.*.:    :        .:* :*    .

       ERESLEVRMREVEEENRALRRQLSLAQGRAPSHRRGNHSKTYSMEEGTGDSENLRAGIVA 120
       -------LM------------------PRVPPDGRLRRAAAL--------DGDPGAGPGD 78
               *                  *.* . * .:: :          . :  **

       GNSSECGQQP-VVEKCETIHVAIVCAGYNASRDVVTLVKSVLFHRRNPLHFHLIADSIAE 179
       HNRSDCGPQPPPPPKCELLHVAIVCAGHNSSRDVITLVKSMLFYRKNPLHLHLVTDAVAR 138
        * *:** **    *** :********:*:****:*****:**:*:****:**::*::*.

       QILATLFQTWMVPAVRVDFYNADELKSEVSWIPNKHYSGIYGLMKLVLTKTLPANLERVI 239
       NILETLFHTWMVPAVRVSFYHADQLKPQVSWIPNKHYSGLYGLMKLVLPSALPAELARVI 198
       :** ***:*********.**:**:** :***********:********  .:***:* ***

       VLDTDITFATDIAELWAVFHKFKGQQVLGLVENQSDWYLGNLWKNHRPWPALGRGYNTGV 299
       VLDTDVTFASDISELWALFAHFSDTQAIGLVENQSDWYLGNLWKNHRPWPALGRGFNTGV 258
       *****:***:**:****:* :*..  *.:**************************:****

       ILLLLDKLRKMKWEQMWRLTAERELMGMLSTSLADQDIFNAVIKQNPFLVYQLPCFWNVQ 359
       ILLRLDRLRQAGWEQMWRLTARRELLSLPATSLADQDIFNAVIKEHPGLVQRLPCVWNVQ 318
       *** **:**:  *********.***:.: :**************::* ** :***.****

       LSDHTRSEQCYRDVSDLKVIHWNSPKKLRVKNKHVEFFRNLYLTFLEYDGNLLRRELFGC 419
       LSDHTLAERCYSEASDLKVIHWNSPKKLRVKNKHVEFFRNFYLTFLEYDGNLLRRELFVC 378
       *****  :*:** :.******************:***************** *

       PSEADVNSENLQKQLSELDEDDLCYEFRRERFTVHRTHLYFLHYEYEPAADSTDVTLVAQ 479
       PSQPPPGAEQLQQALAQLDEEDPCFEFRQQQLTVHRVHVTFLPHEPP-PPRPHDVTLVAQ 437
       **:    .:*:**: *::***:* *:***:::****.*: ** :*         *******

       LSMDRLQMLEAICKHWEGPISLALYLSDAEAQQFLRYAQGSEVLMSRHNVGYHIVYKEGQ 539
       LSMDRLQMLEALCRHWPGPMSLALYLTDAEAQQFLHFVEASPVLAARQDVAYHVVYREGP 497
       ***********:*:** **:******:********::.:.* ** :*::*.**:**:**

       FYPVNLLRNVAMKHISTPYMFLSDIDFLPMYGLYEYLRKSVIQLDLANTK-KAMIVPAFE 598
       LYPVNQLRNVALAQALTPYVFLSDIDFLPAYSLYDYLRASIEQLGLGSRRKAALVVPAFE 557
       :**** *****:  :  ***:******** *.**:*** *: **.*.. :   *::*****

       TLRYRLSFPKSKAELLSMLDMGTLFTFRYHVWTKGHAPTNFAKWRTATTPYRVEWEADFE 658
       TLRYRFSFPHSKVELLALLDAGTLYTFRYHEWPRGHAPTDYARWREAQAPYRVQWAANYE 617
       *****:***:**.***::** ***:***** * :*****::*:** * :****:* *::*

       PYVVVRRDCPEYDRRFVGFGWNKVAHIMELDVQEYEFIVLPNAYMIHMPHAPSFDITKFR 718
       PYVVVPRDCPRYDPRFVGFGWNKVAHIVELDAQEYELLVLPEAFTIHLPHAPSLDISRFR 677
       ***** ****.** *************:***.****::***:*: **:******:**::**

       SNKQYRICLKTLKEEFQQDMSRRYGFAALKYLTAENNS------ 756
       SSPTYRDCLQALKDEFHQDLSRHHGAAALKYLPALQQPQSPARG 721
       *.  ** **::**:**:**:**:**::* ****** * ::
```

Supplementary Fig. 4. Sequence comparison between human LARGE1 and LARGE2. Identical residues are noted by an asterisk (*); similar residues are denoted by a colon (:) or period (.), non-conserved residues are denoted by a space, and indels are noted by a dash (-).

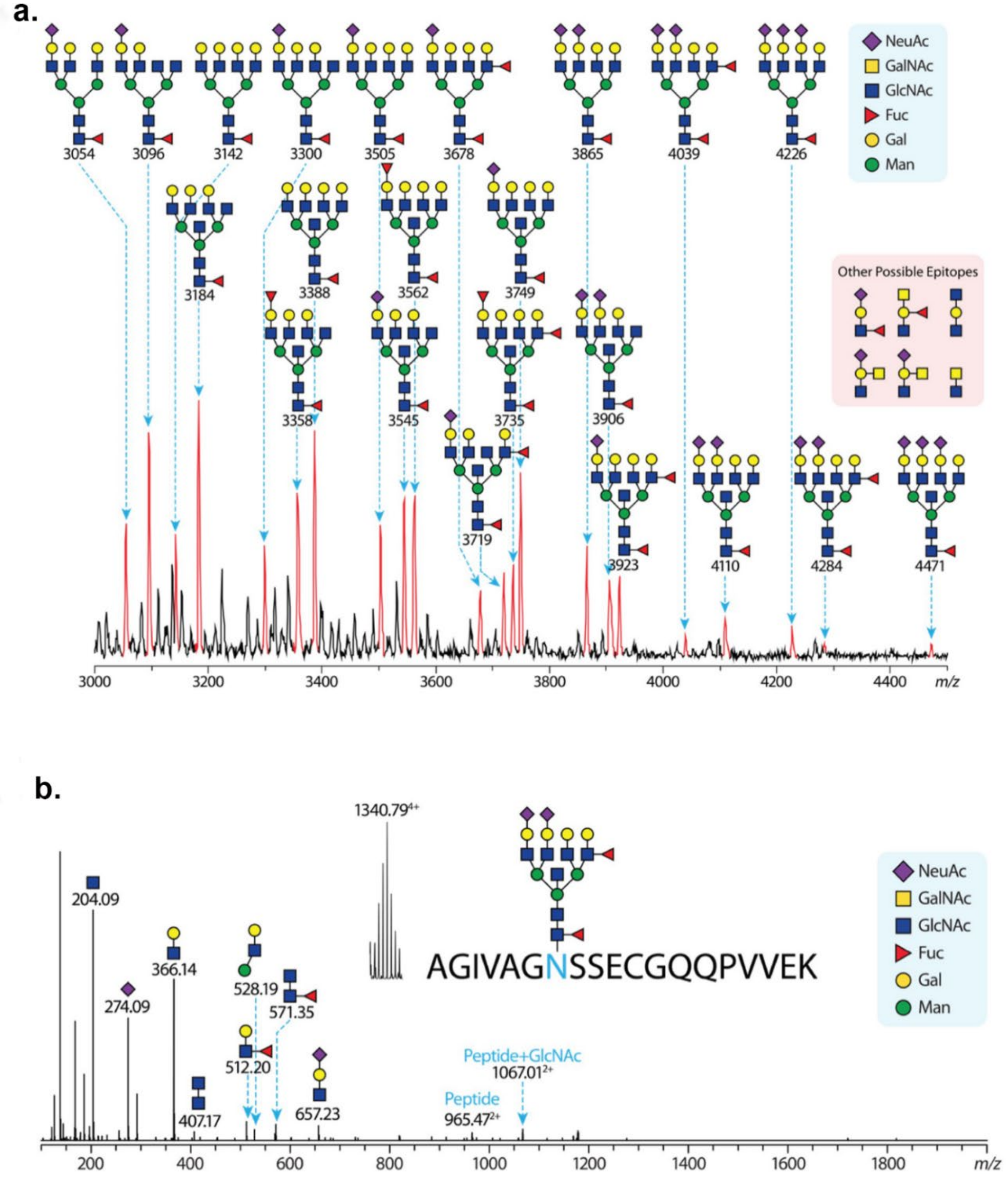

Supplementary Fig. 5. Mass spectrometry (MS) analysis of LARGE1dTM N-glycosylation (N-glycopeptide). a. Glycoproteomics of LARGE1dTM. **b.** A representative MS-MS spectrum of LARGE1dTM N glycopeptide. Structural annotation of glycan is not unambiguous. MALDI-TOF profiling of complex N-glycans released from LARGE1dTM. Sodiated permethylated N-glycans were observed. Structural annotation is not unambiguous and is in favor of more commonly known N-glycan structures.

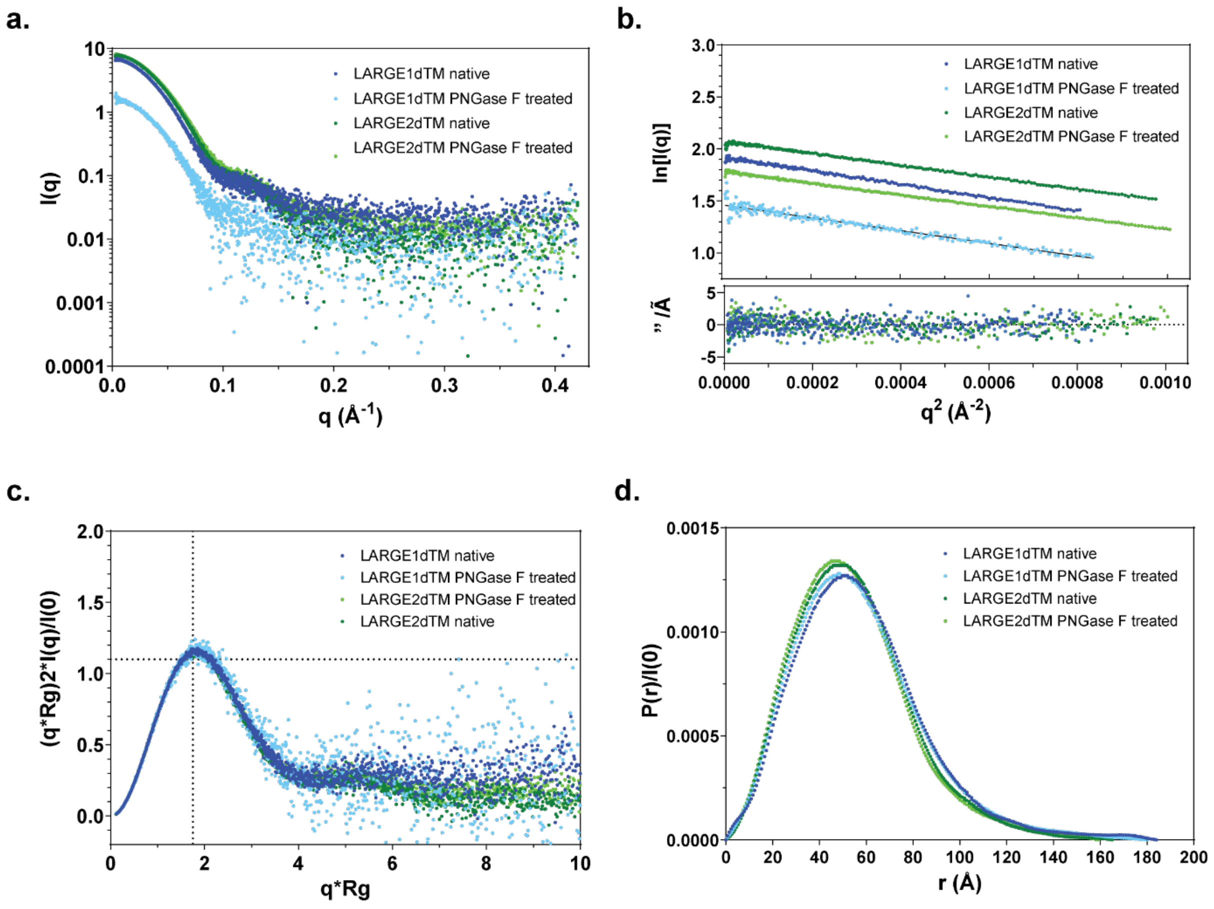

Supplementary Fig. 6. Small-angle X-ray Scattering (SAXS) data for LARGEdTM proteins. **a.** Scattering intensity (I) over radial distance (q). **b.** Guinier plots showing linear distribution at low-q (upper panel) and residuals (lower panel), suggesting a lack of interparticle interference. **c**. Normalized Kratky plot showing globular well-folded entities. **d.** Histogram of interatomic distance vectors. The maximum dimension is ~160–180 Å.

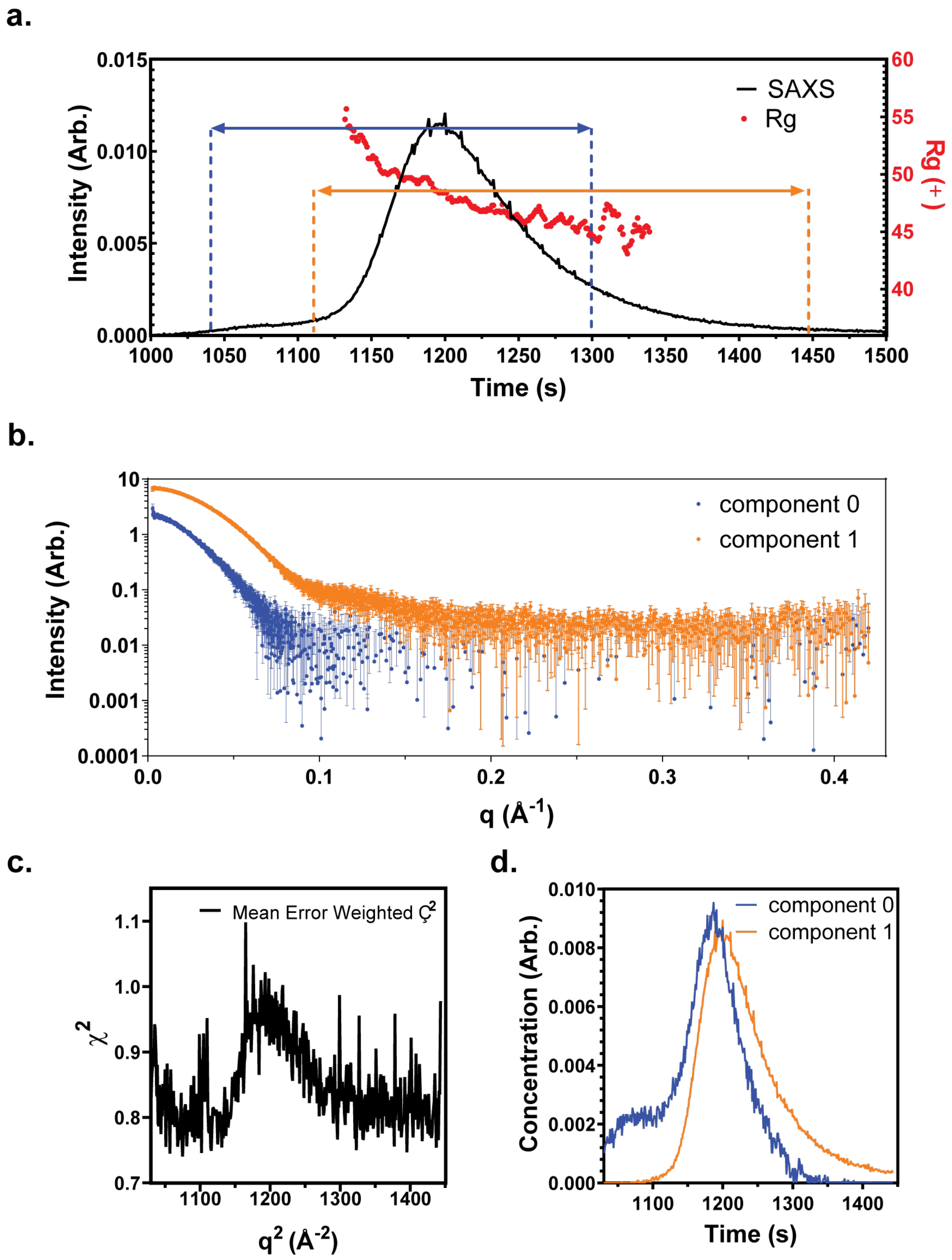

Supplementary Fig. 7. Small-angle X-ray Scattering (SAXS) evolving factor analysis (EFA) of LARGE1dTM. a. The buffer-subtracted integrated SAXS intensity (left axis, in arbitrary scale) and calculated R_g (right axis) as a function of time for the SEC-SAXS experiment. The regions denoted by dashed lines and arrows were deconvoluted by EFA. **b.** Scattering profiles for components determined by EFA. **c.** Mean error-weighted χ^2 of the EFA deconvolution. **d.** Area-normalized component concentration profiles determined by EFA. Colors correspond to component colors on other panels.

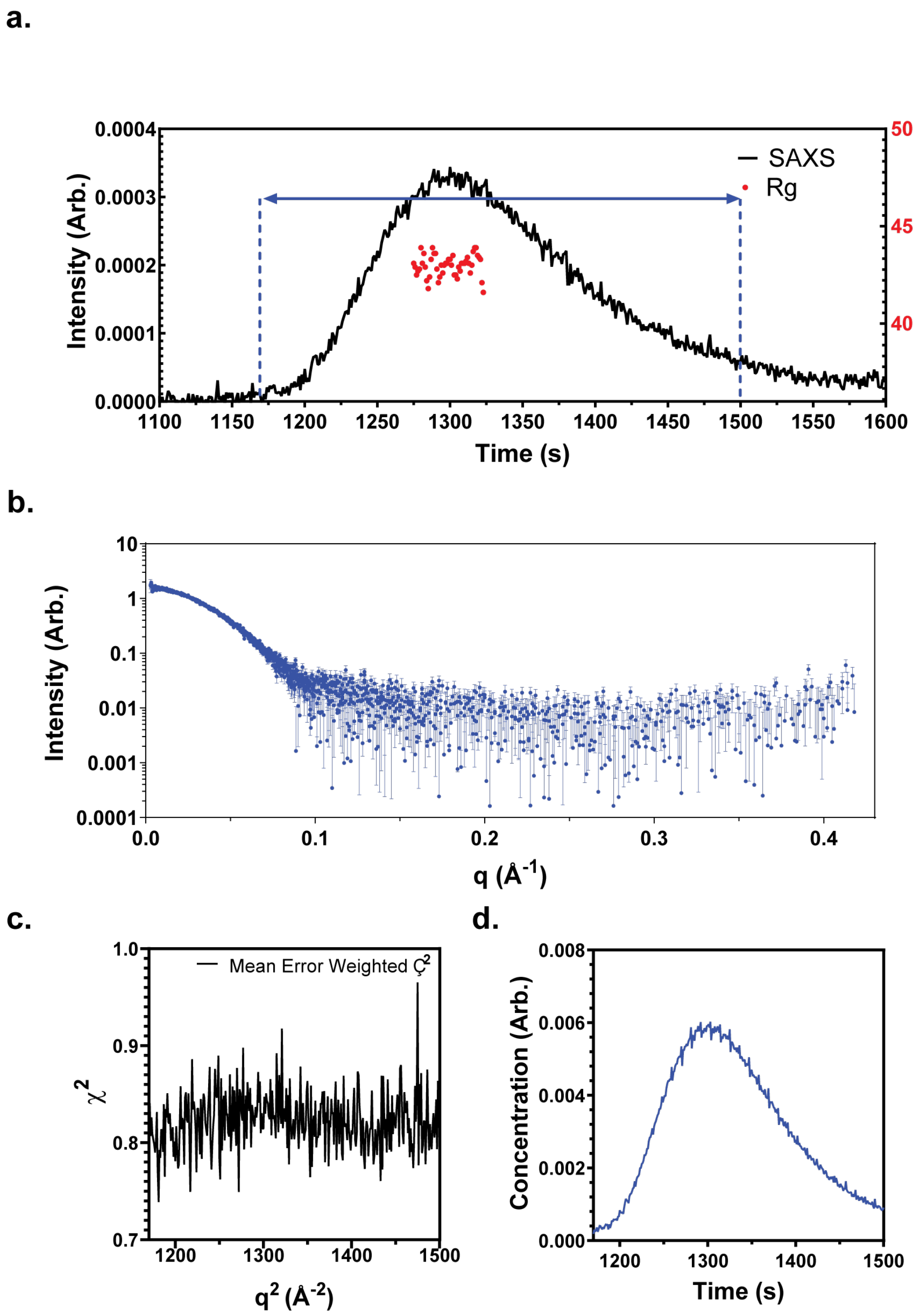

Supplementary Fig. 8. Small-angle X-ray Scattering (SAXS) evolving factor analysis (EFA) of LARGE1dTM treated with PNGase F. a. The buffer-subtracted integrated SAXS intensity (left axis, in arbitrary scale) and calculated R_g (right axis) as a function of time for the SEC-SAXS experiment. The regions denoted by dashed lines and arrows were deconvoluted by EFA. **b.** Scattering profiles for components determined by EFA. **c.** Mean error-weighted χ^2 of the EFA deconvolution. **d.** Area-normalized component concentration profiles determined by EFA. Colors correspond to component colors on other panels.

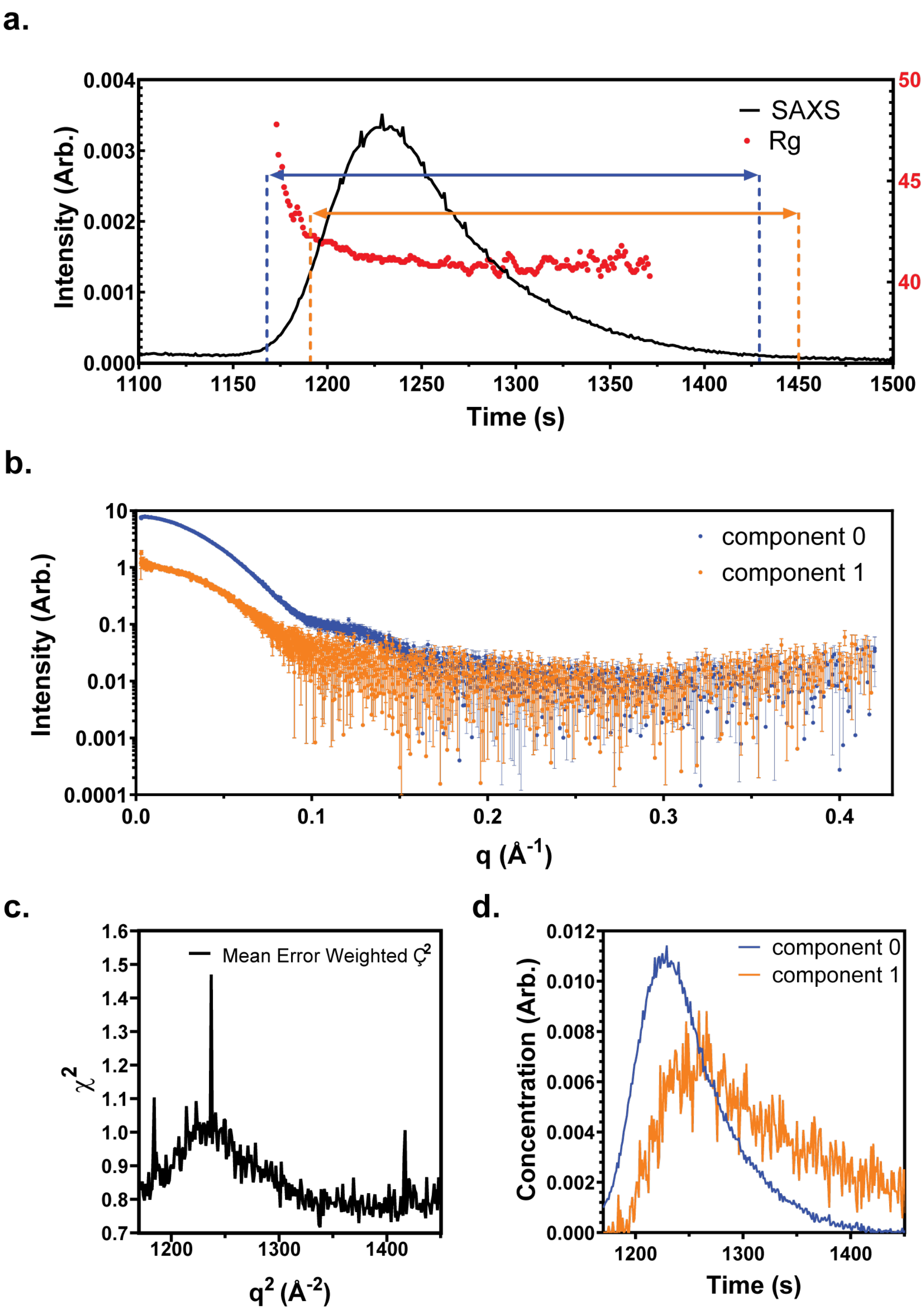

Supplementary Fig. 9. Small-angle X-ray Scattering (SAXS) evolving factor analysis (EFA) of LARGE2dTM. a. The buffer-subtracted integrated SAXS intensity (left axis, in arbitrary scale) and calculated R_g (right axis) as a function of time for the SEC-SAXS experiment. The regions denoted by dashed lines and arrows were deconvoluted by EFA. **b.** Scattering profiles for components determined by EFA. **c.** Mean error-weighted χ^2 of the EFA deconvolution. **d.** Area-normalized component concentration profiles determined by EFA. Colors correspond to component colors on other panels.

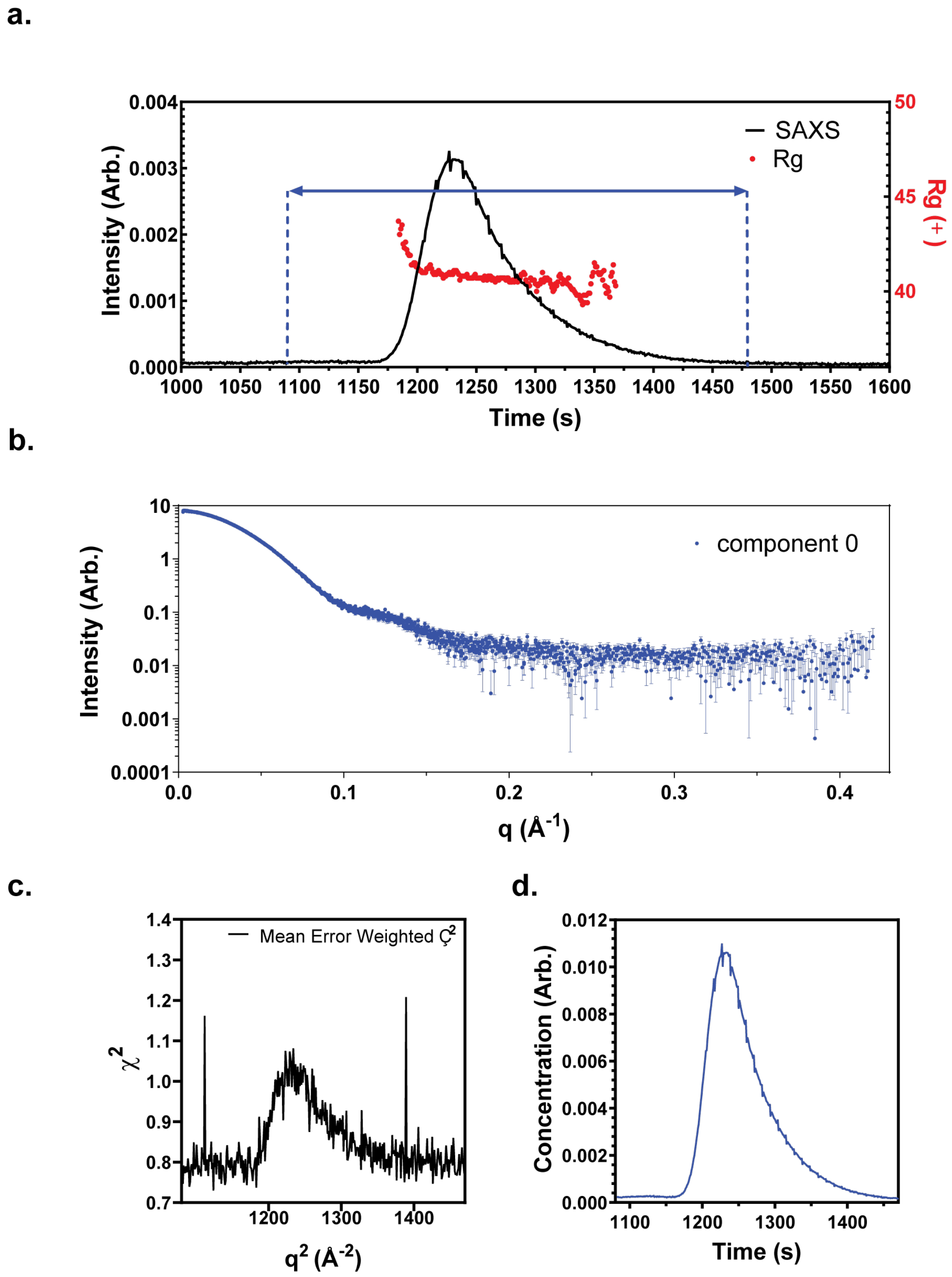

Supplementary Fig. 10. Small-angle X-ray Scattering (SAXS) evolving factor analysis (EFA) of LARGE2dTM treated with PNGase F. a. The buffer subtracted integrated SAXS intensity (left axis, in arbitrary scale) and calculated R_g (right axis) as a function of time for the SEC-SAXS experiment. The regions denoted by dashed lines and arrows were deconvoluted by EFA. **b.** Scattering profiles for components determined by EFA. **c.** Mean error-weighted χ^2 of the EFA deconvolution. **d.** Area-normalized component concentration profiles determined by EFA. Colors correspond to component colors on other panels.

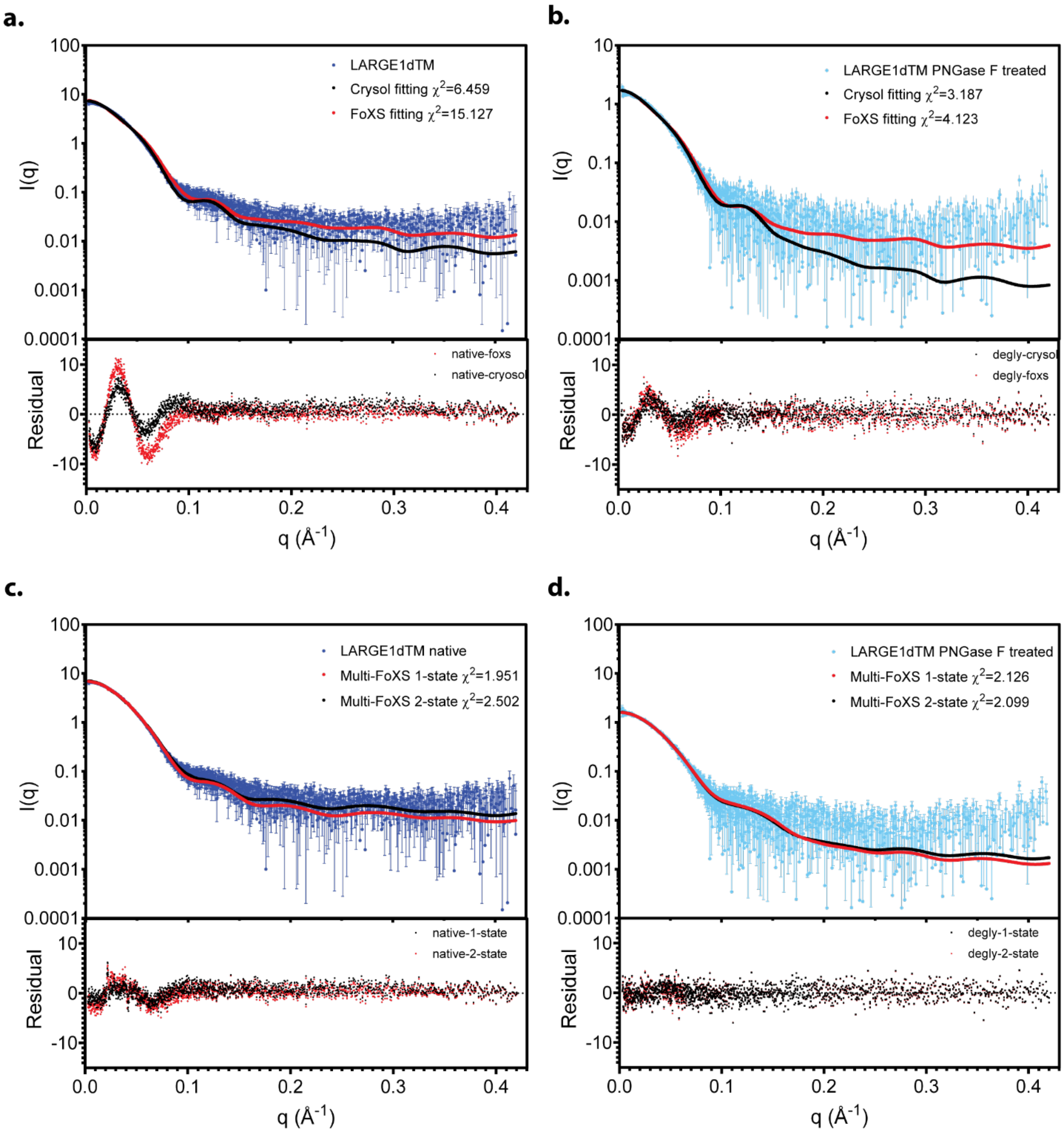

Supplementary Fig. 11. LARGE1dTM cryo-EM models fitted into SAXS data. LARGE1dTM model from single-particle reconstruction in C2 was used to generate an all-atom model in YASARA, which included the coiled-coil domain, flexible linker and hexahistidine tag but not N-glycans. The all-atom model was fitted using CRYSOL (ATSAS), FoxS server with default parameters and multi-FoXS (10,000 conformations; https://modbase.compbio.ucsf.edu).

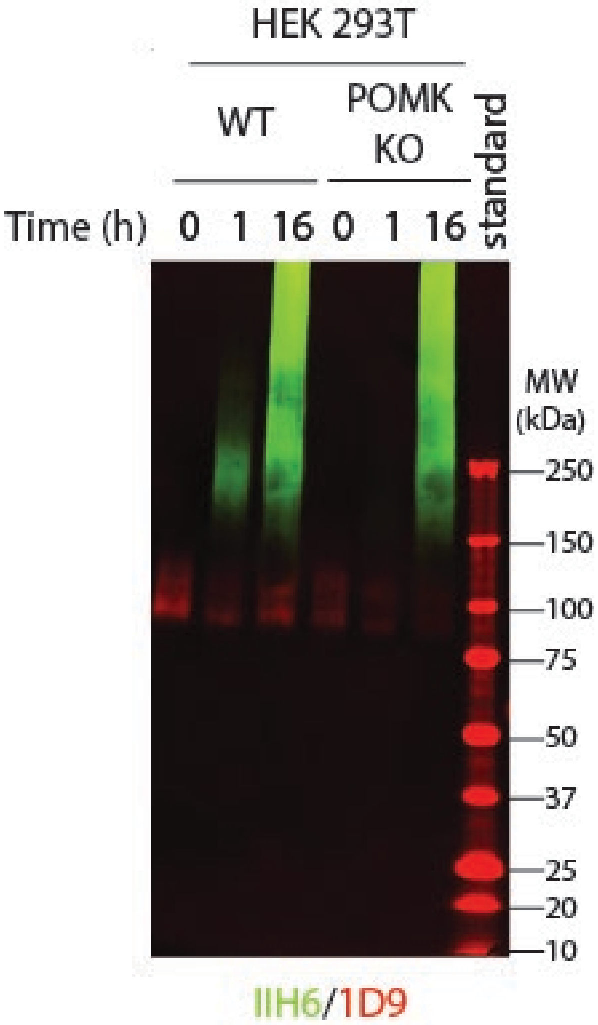

Supplementary Fig. 12. Matriglycan is synthesized inefficiently on prodystroglycan that lacks C6-phosphate on core M3 added by POMK. Anti-matriglycan (IIH6; green) and anti-DGN (1D9; red) Western blot time course of matriglycan polymerized on purified prodystroglycan adenovirally expressed in wild-type or POMK KO HEK 293T cells.

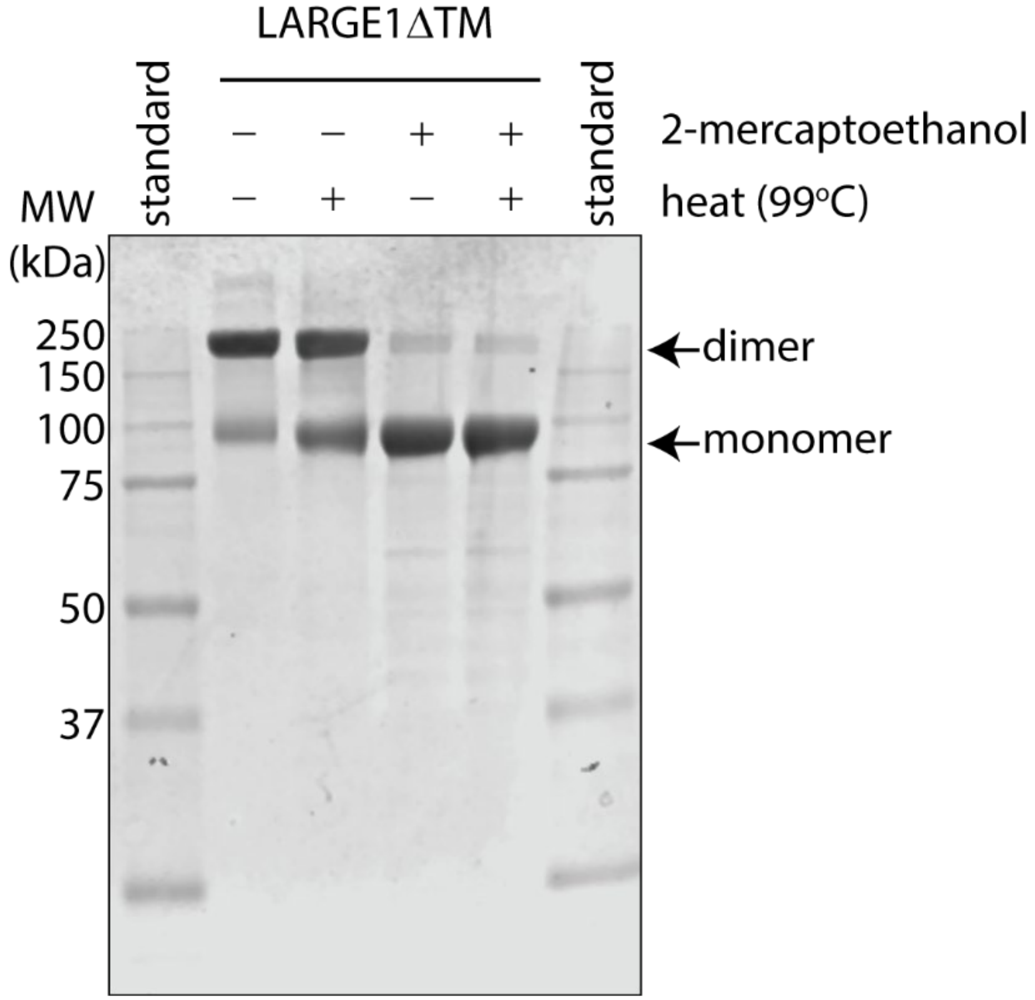

Supplementary Fig. 13. Reducing agent monomerizes partially SDS-resistant LARGE1dTM dimers. SDS-PAGE of LARGE1dTM (LARGE1ΔTM) treated with SDS with or without reducing agent (2-mercaptoethanol) and heated at 99 °C for five minutes. Similar results were obtained independently at least three times.

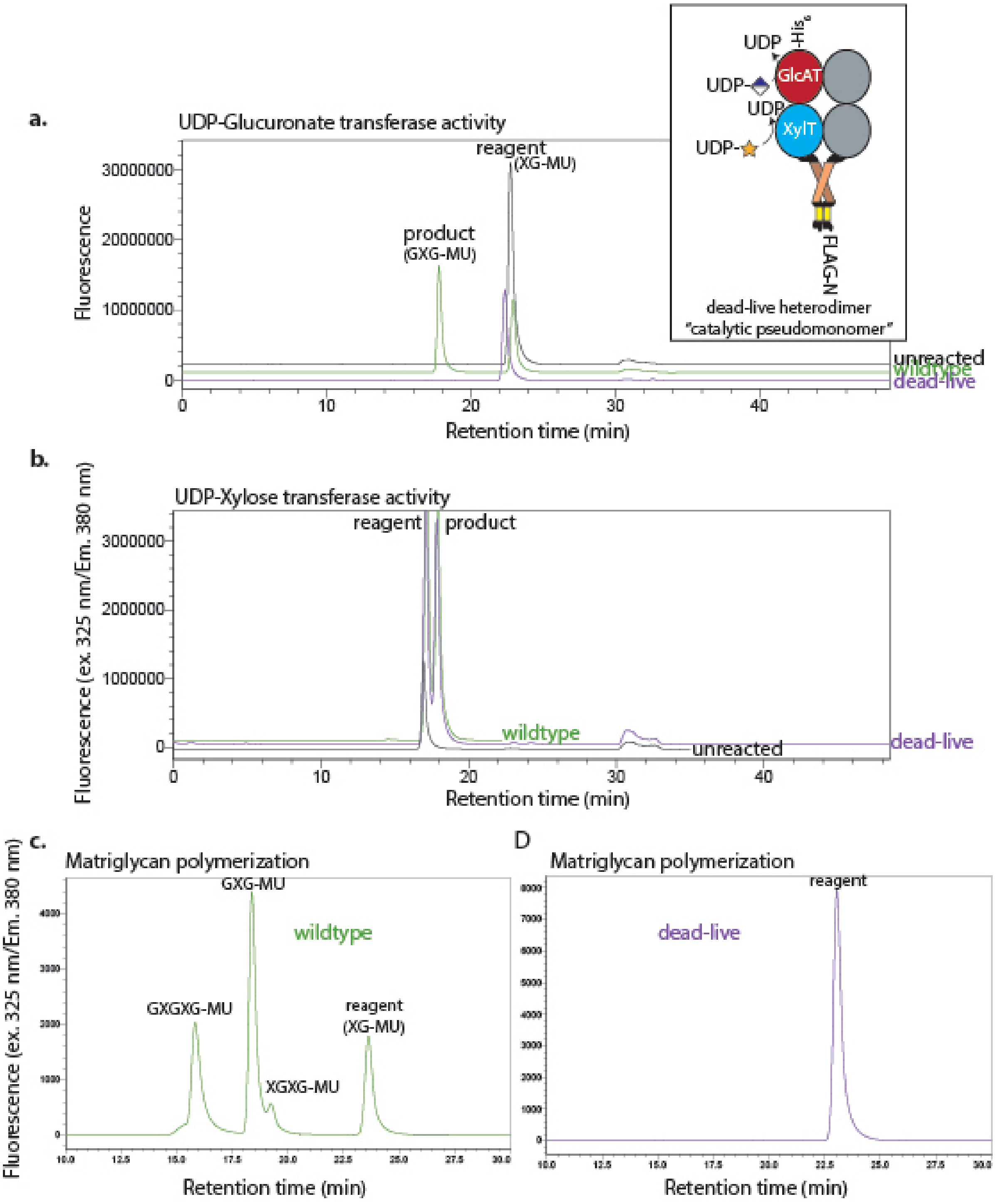

Supplementary Fig. 14. No glucuronic acid transferase activity in half-dead LARGE1dTM. **a.** Glucuronate transfer product is resolved on a C18 reversed-phase chromatogram of pseudomonomeric LARGE1 (dead-live), which are heterodimers that contain an active and an inactive protomer. **b.** Xylose transferase activity is present in a similar assay. **c & d.** Matriglycan can be polymerized by wild-type but not pseudomonomeric LARGE1dTM, suggesting that matriglycan synthesis requires interdomain communication and interprotomer synthesis. Source data are provided as a Source Data file.

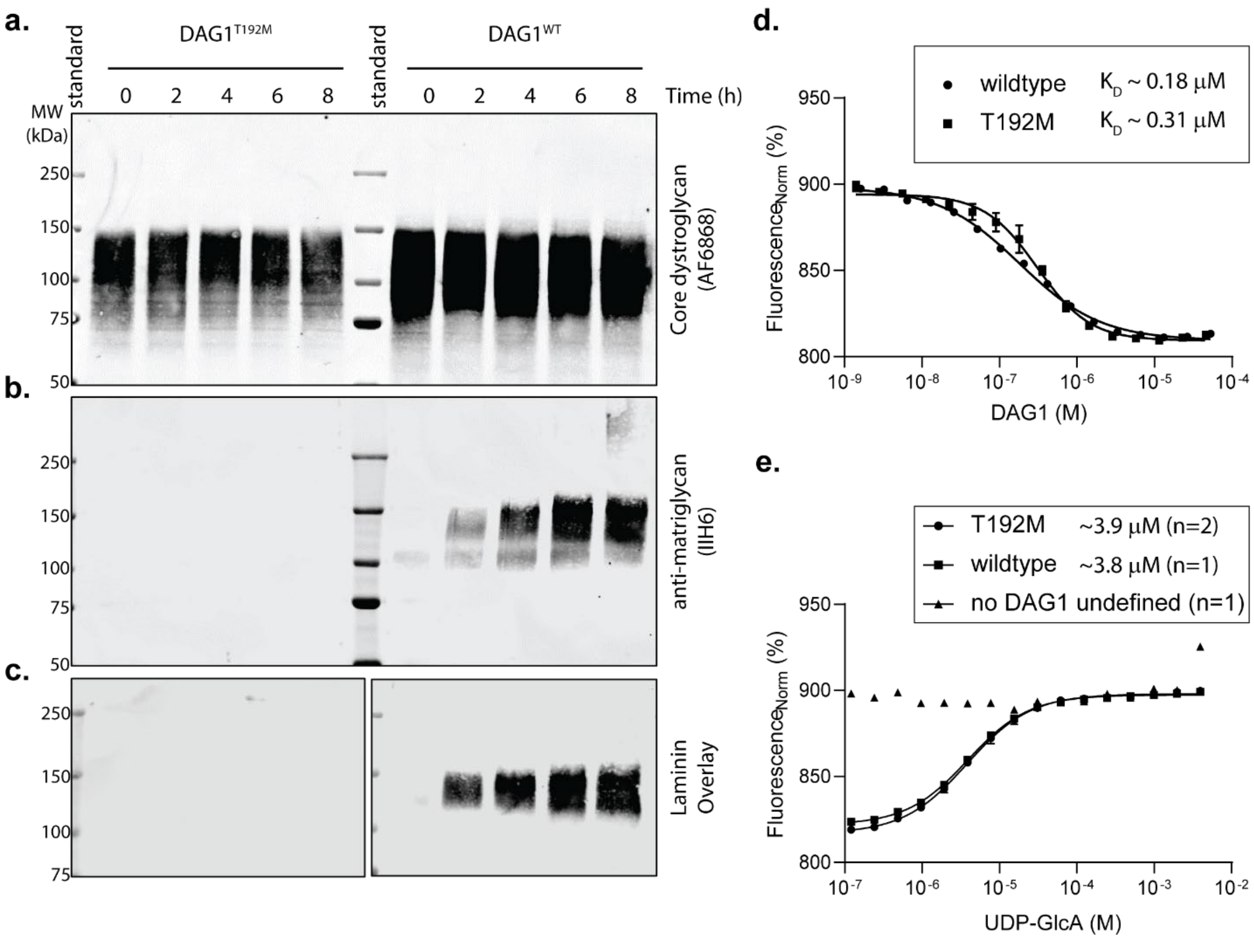

Supplementary Fig. 15. LARGE1dTM binds to but cannot polymerize matriglycan on prodystroglycan^{T192M} *in vitro*. a-c. Western blots (as indicated) of LARGE1 matriglycan synthesis on wild-type and T192M prodystroglycan over time. Similar results were obtained independently at least three times. **d.** Microscale thermophoresis of NHS-red labeled LARGE1dTM titrated with wild-type and T192M prodystroglycan ($DAG1_{28\text{-}340}$) shows that the formation of the enzyme-substrate complex is unaffected. Data points and error bars represent the average and standard error of n=3 replicates, respectively. **e.** Microscale thermophoresis of NHS-red-labeled LARGE1dTM complexed with either wild-type or T192M prodystroglycan and titrated with UDP-glucuronic acid (UDP-GlcA) shows no difference in affinity, suggesting that the inability to polymerize matriglycan is unrelated to binding affinities of mutant and wild-type prodystroglycan for LARGE1dTM. Data points and error bars represent the average and standard error of indicated replicates, respectively. Source data are provided as a Source Data file.

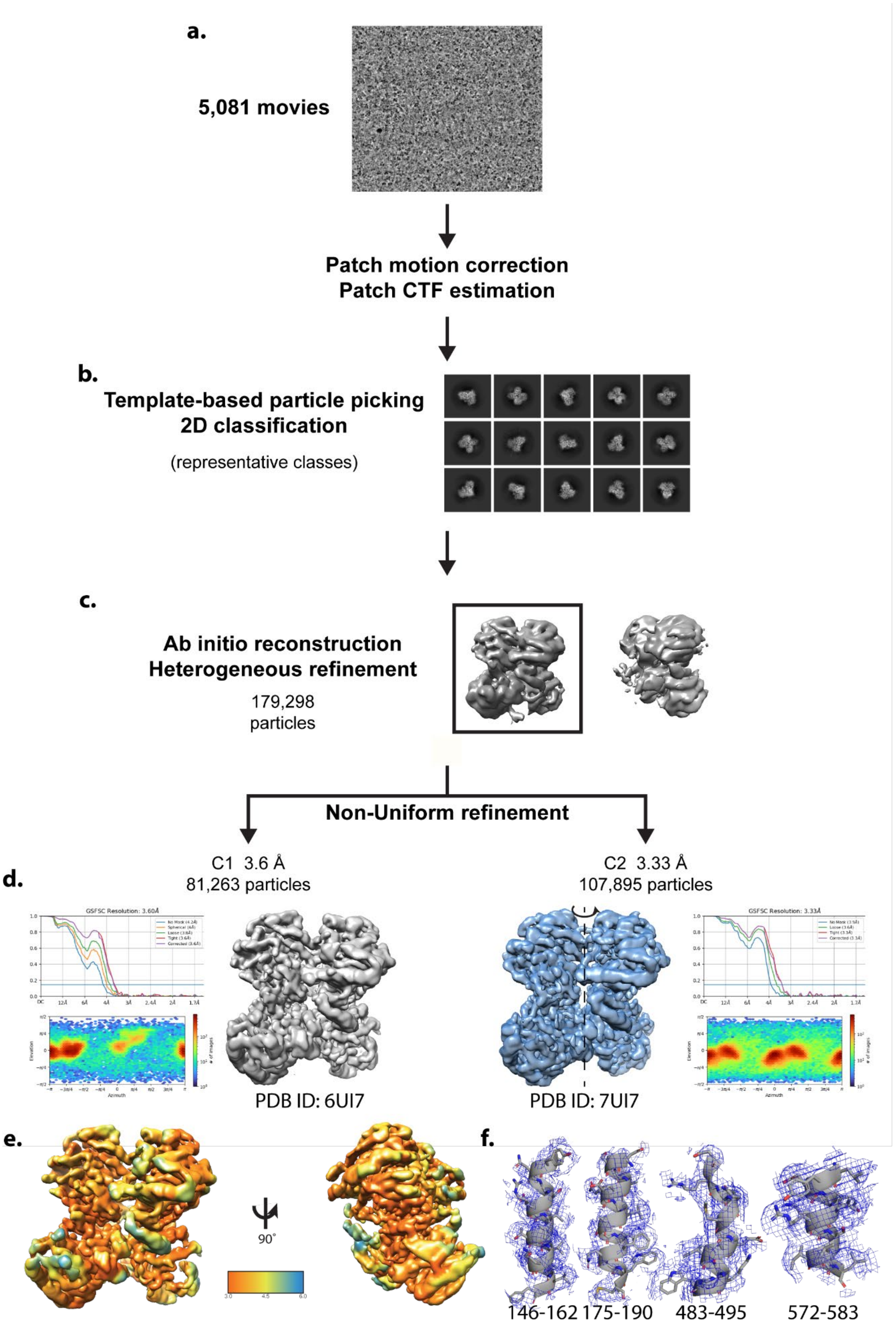
a.
5,081 movies
Patch motion correction
Patch CTF estimation
b.
Template-based particle picking
2D classification
(representative classes)
c.
Ab initio reconstruction
Heterogeneous refinement
179,298
particles
Non-Uniform refinement
d.
C1 3.6 Å
81,263 particles
C2 3.33 Å
107,895 particles
GSFSC Resolution: 3.60Å
GSFSC Resolution: 3.33Å
PDB ID: 6UI7
PDB ID: 7UI7
e.
90°
f.
146-162
175-190
483-495
572-583

Supplementary Fig. 16. LARGE1dTM cryo-EM processing workflow. Cryo-EM reconstruction of LARGE1dTM in CryoSPARC. **a.** Example micrograph. Template-picker was used on motion- and CTF-corrected micrographs of LARGE1dTM. **b.** Sample of selected 2D class averages used for *ab initio* reconstruction. **d.** Particles were non-uniformly refined separately in C1 (no symmetry; PDB ID: 7UI6) and C2 (PDB ID: 7UI7) symmetries. The axis of two-fold rotational symmetry (C2) is by the dotted line on the reconstructed volume on the right-hand-side. A slightly modified workflow resulted in more particles for the C2 reconstruction. **e.** Volume colored by local resolution. **f.** Examples of helices fitted into volumes.

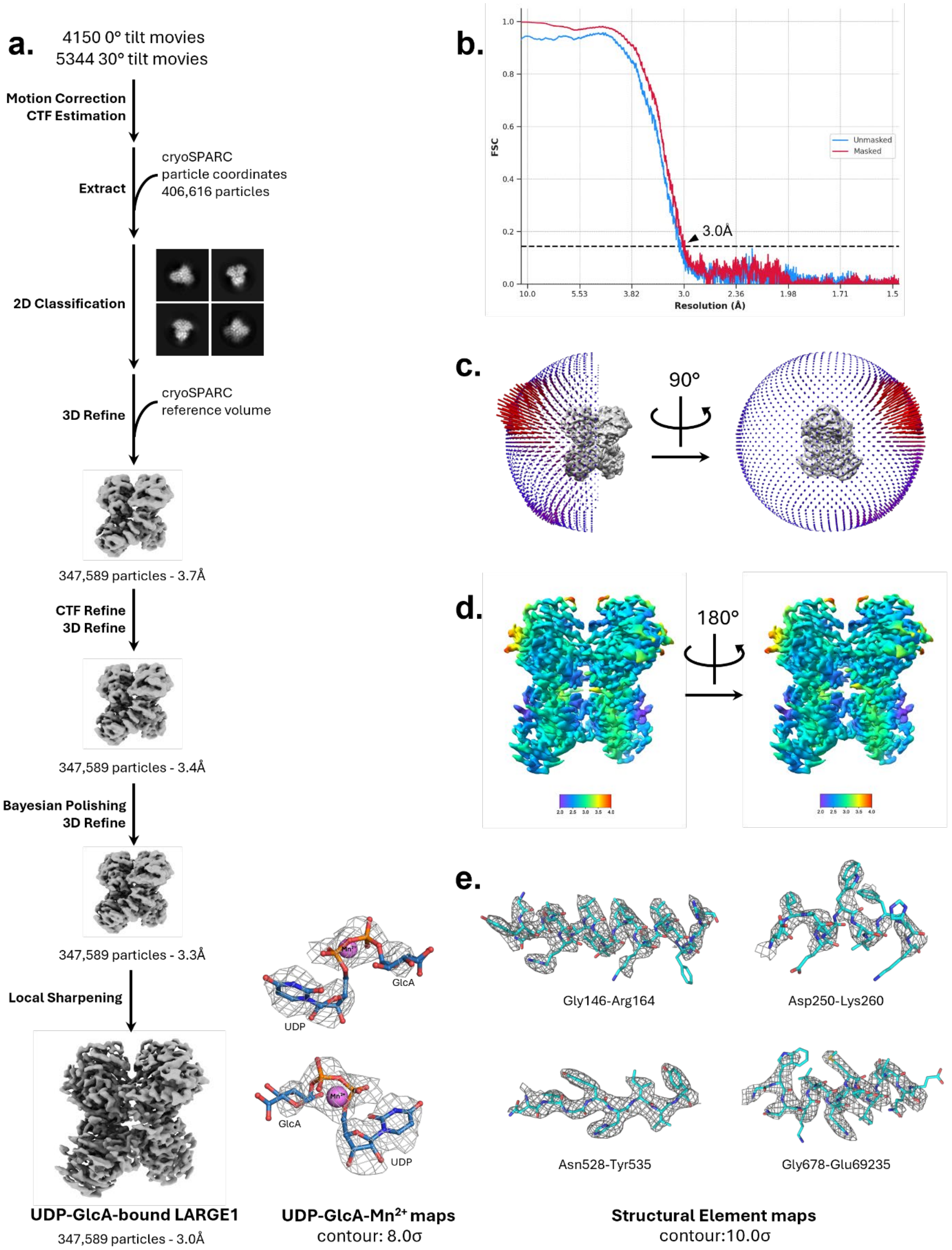
a.
4150 0° tilt movies
5344 30° tilt movies
Motion Correction
CTF Estimation
Extract
cryoSPARC
particle coordinates
406,616 particles
2D Classification
3D Refine
cryoSPARC
reference volume
347,589 particles - 3.7Å
CTF Refine
3D Refine
347,589 particles - 3.4Å
Bayesian Polishing
3D Refine
347,589 particles - 3.3Å
Local Sharpening
UDP-GlcA-bound LARGE1
347,589 particles - 3.0Å
GlcA
UDP
UDP-GlcA-Mn2+ maps
contour: 8.0σ
b.
FSC
Unmasked
Masked
3.0Å
Resolution (Å)
c.
90°
d.
180°
e.
Gly146-Arg164
Asp250-Lys260
Asn528-Tyr535
Gly678-Glu69235
Structural Element maps
contour:10.0σ

Supplementary Fig. 17. LARGE1-DAG1$_{28\text{-}340}$ cryo-EM processing workflow. a. Cryo-EM reconstruction of LARGE1dTM-DAG1$_{28\text{-}340}$ complex in RELION with inputs from CryoSPARC as indicated. **b.** FSC curves for masked and unmasked volumes. **c.** Orientation distribution analysis displays minor preferred orientation. **d.** Volume colored by local resolution. **e.** Example helices fitted into reconstructed volumes.

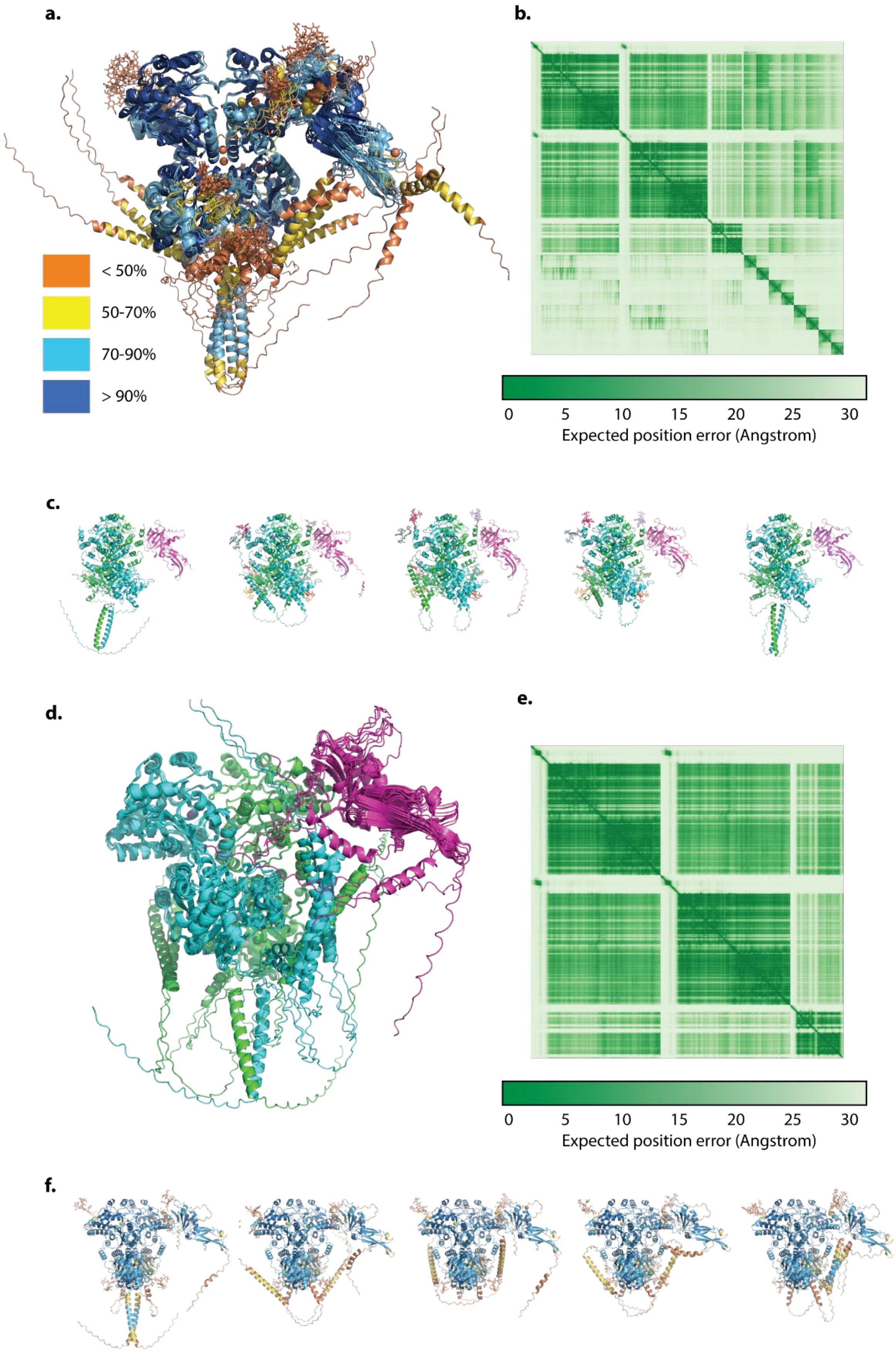
a.
< 50%
50-70%
70-90%
> 90%
b.
0
5
10
15
20
25
30
Expected position error (Angstrom)
c.
d.
e.
0
5
10
15
20
25
30
Expected position error (Angstrom)
f.

Supplementary Fig. 18. AlphaFold 3 models of LARGE1 (residues 31-756; green and cyan cartoon) with prodystroglycan (28-320; magenta cartoon). (**a**) All five models shown in Figure 5c and f are overlaid. (**b**) Predicted aligned error matrix for (**c**) an independent prediction with glycosylation (sticks) and without (**d-f**) tetra-antennary N-glycan modifications (92N, 242N, 724N and 725N with glycan chain NAG(FUC)(NAG(MAN(MAN(NAG)(NAG))(NAG))) with a single chain of prodystroglycan along with four Mn^{2+} ions (violet spheres) and one Ca^{2+} ion (green sphere). (**d**) All five AlphaFold 3 predicted models of LARGE1-prodystroglycan without N-glycan modifications are overlaid. (**e**) Predicted aligned error matrix for **d**. (**f**) All five models are shown separately colored by confidence according to key in (**a**). Supplementary Table 5 shows extended parameters to fulfill the author checklist for structures predicted using AlphaFold 3 Server.

Supplementary Table 1. LARGE1dTM and LARGE2dTM SEC-MALS-SAXS experimental conditions

**a) Sample details**	
SEC Column	Superdex 200 increase 10/300
Loaded concentration (mg/mL)	4-8
Injection volume (µL)	300-500
Flow rate (mL/min)	0.6
Solvent (solvent blanks taken from SEC flowthrough prior to elution of protein)	20 mM HEPES pH 7.4, 150 mM NaCl
**b) SAXS data-collection parameters**	
Instrument	BioCAT facility at the Advanced Photon Source beamline 18ID<br>with Eiger2 XE 9M (Dectris) detector
Wavelength (Å)	1.033
Beam size (μm^2)	150 (h) x 25 (v) focused on the detector
Camera length (m)	3.6
q measurement range ($Å^{-1}$)	0.003-0.42
Absolute scaling method	Glassy Carbon, NIST SRM 3600
Basis for normalization to constant counts	To incident intensity, by ion chamber counter
Monitoring for radiation damage	Automated frame-by-frame comparison of relevant regions using CORMAP (Franke et al., 2015) implemented in BioXTAS RAW
Exposure time	0.5 s exposure time with a 1 s total exposure period (0.5 s on, 0.5 s off) of entire SEC elution
Sample configuration	SEC-MALS-SAXS. Size separation used a Superdex 200 Increase 10/300 GL column and a 1260 Infinity II HPLC (Agilent Technologies). UV data were measured using the HPLC, and MALS-DLS-RI data by DAWN HELEOS-II (17 MALS + 1 DLS channels) and Optilab T-rEX (RI) instruments (Wyatt Technology). SAXS data was measured in a sheath-flow cell with an effective path length of 0.542 mm.
Sample temperature (ºC)	23
**c) Software employed for SAXS data reduction, analysis, and interpretation**	
SAXS data reduction	Radial averaging; frame comparison, averaging, and subtraction done using BioXTAS RAW 2.1.1 (Hopkins et al., 2017)
Basic analysis: Guinier, MW, Normalized Kratky, P(r)	Guinier fit and M.W. using BioXTAS RAW, P(r) function using GNOM (Svergun, 1992). RAW uses MoW and Vc M.W. methods (Rambo & Tainer, 2013; Piiadov et al., 2018)
MALS-DLS-RI analysis	Astra 8 (Wyatt)

Supplementary Table 2. Cryo-EM data collection parameters

	**LARGE1 C1 symmetry**	**LARGE1 C2 symmetry**	**LARGE1-DAG1$_{28\text{-}340}$**
Data collection and image processing			
Microscope	Titan Krios	Titan Krios	Titan Krios
Voltage (kV)	300	300	300
Camera	K3	K3	K3
Magnification	29,000 x	29,000 x	105,000 x
Electron exposure (e^-/Å^2)	50	50	50
Exposure time (s)	1.66	1.66	3.795
Number of frames	50	50	50
Defocus range (μm)	-0.8 to -2.0	-0.8 to -2.0	-0.8 to 0.2
Super resolution pixel size (Å)	0.40075	0.40075	0.827
Number of movies (tilt)	5,085 (0°)	5,085 (0°)	4150 (0°)<br>5344 (30°)
Particles for final reconstruction (no.)	81,263	107,895	347, 589
Map resolution (Å)<br>Half map FSC threshold	3.7<br>0.143	3.4<br>0.143	3.0<br>0.143
EMDB accession code	26540	26541	26541
Model refinement statistics			
Model composition<br>Non-hydrogen atoms<br>Protein residues<br>Ligands	 10,148<br>609<br>4	 10,214<br>613<br>4	 9982<br>602<br>4
Cross correlation<br>Mask<br>Volume	 0.58<br>0.59	 0.66<br>0.67	 0.79<br>0.77
RMSD<br>Bond length (Å)<br>Bond Angles (°)	 0.005<br>0.971	 0.004<br>0.612	 0.02<br>0.379
Ramachandran<br>Favored (%)<br>Allowed (%)<br>Outlier (%)	 94.96<br>4.79<br>0.25	 92.36<br>7.64<br>0.00	 97.32<br>2.68<br>0
Validation<br>MolProbity score<br>Clashscore<br>Rotamer outliers (%)	 2.15<br>18.99<br>1.09	 2.13<br>14.09<br>0.00	 1.44<br>5.73<br>0
PDB accession code	7UI6	7UI7	9E1T

Supplementary Table 3. LARGEdTM SAXS parameters

**(a) Structural parameters**				
	LARGE1dTM	LARGE1dTM PNGase F treated	LARGE2dTM	LARGE2dTM PNGase F treated
Guinier Analysis				
I(0) (Arb.)	6.818 ± 0.007	1.584 ± 0.004	7.951 ± 0.005	8.018 ± 0.004
R_g (Å)	43.99 ± 0.08	42.83 ± 0.12	41.56 ± 0.05	40.87 ± 0.03
q-range ($Å^{-1}$)	0.00391-0.02946	0.00428-0.03033	0.00279-0.0312	0.00279-0.03169
$q_{max}R_g$	1.2959	1.2991	1.2966	1.2952
Coefficient of correlation, r^2	0.9973	0.9752	0.9957	0.9984
Volume ($Å^3$ adjusted V_P as SAXS MoW2)	264425	248999	256585	230571
MW, MoW2 method (kDa)	219 (1.22)	206 (1.15)	212 (1.26)	191 (1.13)
MW, Vc method (kDa)	173 (0.96)	173 (0.96)	184 (1.09)	161 (0.96)
P(*r*) analysis				
I(0) (Arb.)	6.845 ± 0.010	1.595 ± 0.005	7.973 ± 0.006	8.048 ± 0.004
R_g (Å)	45.05 ± 0.16	44.22 ± 0.29	42.18 ± 0.08	41.66 ± 0.04
D_{max} (Å)	184	180	165	159
q-range ($Å^{-1}$)	0.00391-0.4202	0.00428-0.4202	0.00279-0.4202	0.00279-0.4202
χ^2 (total estimate from GNOM)	0.907 (0.711)	2.078 (0.7647)	1.051 (0.7729)	1.641 (0.8151)

**(b) Shape modelling fitting parameters**				
DAMMIF (Slow mode, default parameters, 15 calculations				
q-range for fitting ($Å^{-1}$)	0.00391-0.18185	0.00428-0.18669	0.00279-0.19258	0.00279-0.19587
Symmetry, anisotropy assumptions	P1, unknown	P1, unknown	P1, unknown	P1, unknown
Ambiguity score (AMBIMETER)	0.4771	1.505	0.4771	0.4771
NSD (standard deviation), No. of clusters	0.805 (0.042), 1	0.834 (0.053), 1	0.690 (0.074), 1	0.703 (0.061), 1
χ^2 range	1.401-1.476	4.258-4.343	2.491-2.951	6.646-7.315
Model MW estimate range (ratio to expected)	216.4 (1.21)	239.8 (1.34)	242.1 (1.44)	221.5 (1.32)
Model R_g range	45.161-45.186	46.39-46.42	42.618-42.644	42.186-42.29
Model D_{max} range	180-202	179-215	167-185	159.8-182
Resolution (SASRES) (Å)	59 ± 4	61 ± 4	45 ± 3	45 ± 3
DAMMIN (Refinement of damstart.pdb)				
q-range for fitting ($Å^{-1}$)	0.00391-0.18185	0.00428-0.18669	0.00279-0.19258	0.00279-0.19587
Symmetry, anisotropy assumptions	P1, unknown	P1, unknown	P1, unknown	P1, unknown
Ambiguity score (AMBIMETER)	0.4771	1.505	0.4771	0.4771
χ^2	1.43	4.359	2.716	7.365
Model MW estimate (ratio to expected)	271.9 (1.52)	232.9 (1.30)	230.4 (1.37)	213.8 (1.27)
Model R_g range	45.2	46.36	42.62	42.29
Model D_{max} range	183.5	173.5	167.2	159.8

Supplementary Table 4. SAXS fitting parameters

**(a) Fitting parameters**		
	LARGE1dTM	LARGE1dTM PNGase F treated
1.Crysol fitting		
Excluded volume (Å^3)	224,557	224,557
Rg (Å)	51.65	51.65
χ^2	6.459	3.187
2.FoXS fitting		
Rg (Å)	52.2	52.2
c1	1.05	1.05
c2	0.28	-0.53
χ^2	15.127	4.123
3.Multi-FoXS fitting		
Best scoring 1-state model		
c1	1.04	1.01
c2	0.27	-0.50
χ^2	1.951	2.126
Best scoring 2-state model		
c1	1.05	1.03
c2	-0.05	-0.50
χ^2	2.502	2.099
model 1 Rg (Å)	46.27	43.88
w1	0.752	0.793
model 2 Rg (Å)	44.68	49.61
w2	0.248	0.207

Supplementary Table 5. AlphaFold parameters and outputs for dimeric LARGE1 with DGN

Instance (date)	1 (2024-05-15)	2 (2025-02-27)	3 (2025-02-27)
LARGE1 residues × n	**O95461 (1-756) × 2**	**O95461 (31-756) × 2**	**O95461 (31-756) × 2**
DAG1	**Q14118 (28-320) × 1**	**Q14118 (28-320) × 1**	**Q14118 (28-320) × 1**
>sp|O95461|LARG1_HUMAN Xylosyl- and glucuronyltransferase LARGE1 OS=Homo sapiens OX=9606 GN=LARGE1 PE=1 SV=1<br>MLGICRGRRKFLAASLSLLCIPAITWIYLFSGSFEDGKPVSLSPLESQAHSPRYTASSQRERESLEVRMREVEEENRALRRQLSLAQGRAPSHRRGNHSKTYSME<br>EGTGDSENLRAGIVAGNSSECGQQPVVEKCETIHVAIVCAGYNASRDVVTLVKSVLFHRRNPLHFHLIADSIAEQILATLFQTWMVPAVRVDFYNADELKSEVSW<br>IPNKHYSGIYGLMKLVLTKTLPANLERVIVLDTDITFATDIAELWAVFHKFKGQQVLGLVENQSDWYLGNLWKNHRPWPALGRGYNTGVILLLLDKLRKMKWEQM<br>WRLTAERELMGMLSTSLADQDIFNAVIKQNPFLVYQLPCFWNVQLSDHTRSEQCYRDVSDLKVIHWNSPKKLRVKNKHVEFFRNLYLTFLEYDGNLLRRELFGCP<br>SEADVNSENLQKQLSELDEDDLCYEFRRERFTVHRTHLYFLHYEYEPAADSTDVTLVAQLSMDRLQMLEAICKHWEGPISLALYLSDAEAQQFLRYAQGSEVLMS<br>RHNVGYHIVYKEGQFYPVNLLRNVAMKHISTPYMFLSDIDFLPMYGLYEYLRKSVIQLDLANTKKAMIVPAFETLRYRLSFPKSKAELLSMLDMGTLFTFRYHVW<br>TKGHAPTNFAKWRTATTPYRVEWEADFEPYVVVRRDCPEYDRRFVGFGWNKVAHIMELDVQEYEFIVLPNAYMIHMPHAPSFDITKFRSNKQYRICLKTLKEEFQ<br>QDMSRRYGFAALKYLTAENNS			
>sp|Q14118|DAG1_HUMAN Dystroglycan 1 OS=Homo sapiens OX=9606 GN=DAG1 PE=1 SV=2<br>MRMSVGLSLLLPLSGRTFLLLLSVVMAQSHWPSEPSEAVRDWENQLEASMHSVLSDLHEAVPTVVGIPDGTAVVGRSFRVTIPTDLIASSGDIIKVSAAGKEALP<br>SWLHWDSQSHTLEGLPLDTDKGVHYISVSATRLGANGSHIPQTSSVFSIEVYPEDHSELQSVRTASPDPGEVVSSACAADEPVTVLTVILDADLTKMTPKQRIDL<br>LHRMRSFSEVELHNMKLVPVVNNRLFDMSAFMAGPGNAKKVVENGALLSWKLGCSLNQNSVPDIHGVEAPAREGAMSAQLGYPVVGWHIANKKPPLPKRVRRQIH<br>ATPTPVTAIGPPTTAIQEPPSRIVPTPTSPAIAPPTETMAPPVRDPVPGKPTVTIRTRGAIIQTPTLGPIQPTRVSEAGTTVPGQIRPTMTIPGYVEPTAVATPP<br>TTTTKKPRVSTPKPATPSTDSTTTTTRRPTKKPRTPRPVPRVTTKVSITRLETASPPTRIRTTTSGVPRGGEPNQRPELKNHIDRVDAWVGTYFEVKIPSDTFYD<br>HEDTTTDKLKLTLKLREQQLVGEKSWVQFNSNSQLMYGLPDSSHVGKHEYFMHATDKGGLSAVDAFEIHVHRRPQGDRAPARFKAKFVGDPALVLNDIHKKIALV<br>KKLAFAFGDRNCSTITLQNITRGSIVVEWTNNTLPLEPCPKEQIAGLSRRIAEDDGKPRPAFSNALEPDFKATSITVTGSGSCRHLQFIPVVPPRRVPSEAPPTE<br>VPDRDPEKSSEDDVYLHTVIPAVVVAAILLIAGIIAMICYRKKRKGKLTLEDQATFIKKGVPIIFADELDDSKPPPSSSMPLILQEEKAPLPPPEYPNQSVPETT<br>PLNQDTMGEYTPLRDEDPNAPPYQPPPPFTAPMEGKGSRPKNMTPYRSPPPYVPP			
pLDDT score	By color (see below)	By color (see below)	By color (see below)
Seed	736608798	**1483329445**	1860396058
PAE matrix	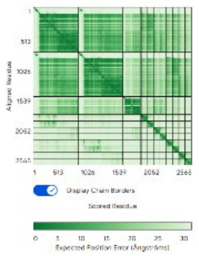	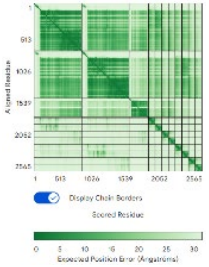	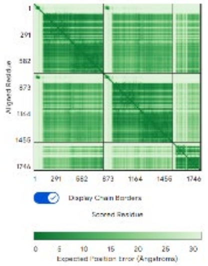

Ions (×n)	Mn^{2+} × 4 and Ca^{2+} × 1	Mn^{2+} × 4 and Ca^{2+} × 1	Mn^{2+} × 4 and Ca^{2+} × 1
Glycosylation residues	92N, 242N, 724N and 725N: NAG(FUC)(NAG(MAN(MAN(NAG)(NAG))(NAG)))	92N, 242N, 724N and 725N: NAG(FUC)(NAG(MAN(MAN(NAG)(NAG))(NAG)))	No PTMs
Viewing angle 1	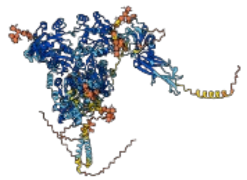	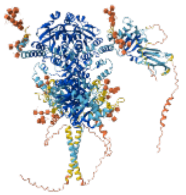	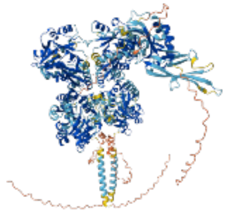
Viewing angle 2	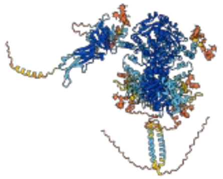	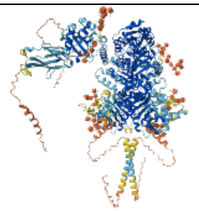	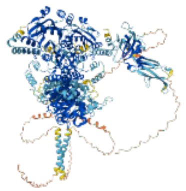

Very high (plDDT > 90) Confident (90 > plDDT > 70) Low (70 > plDDT > 50) Very low (plDDT < 50)

Gel source data for Supplementary Fig. 12

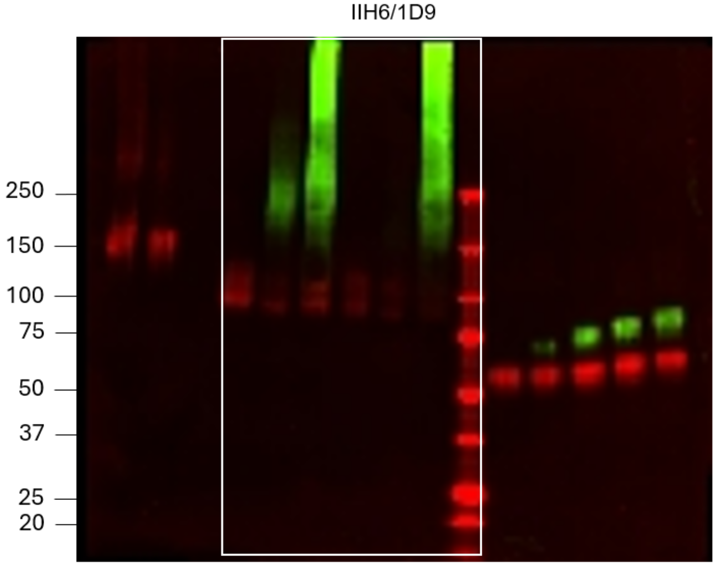

Gel source data for Supplementary Fig. 13

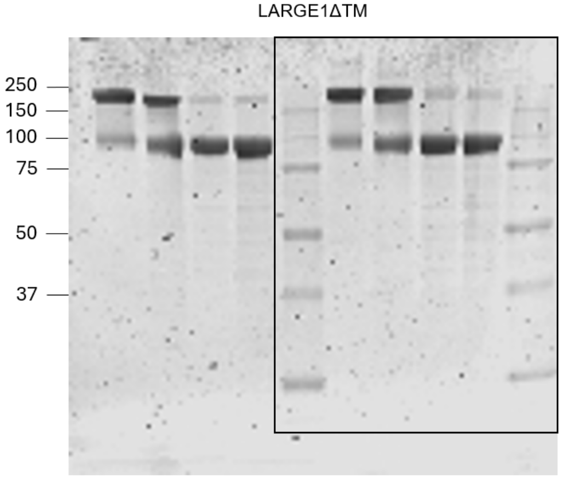

Gel source data for Supplementary Fig. 15a

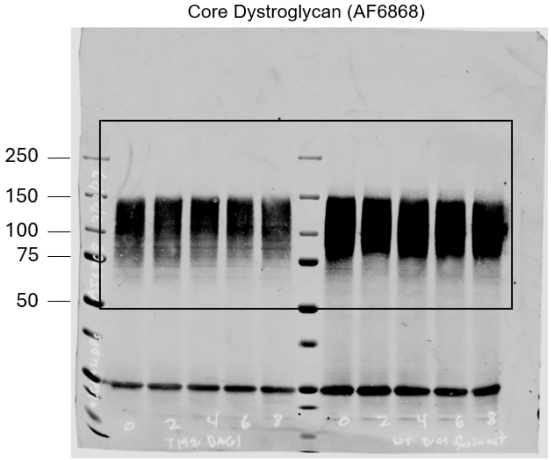

Gel source data for Supplementary Fig. 15b

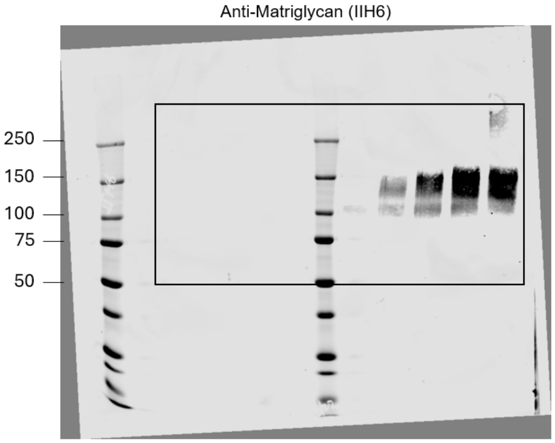

Gel source data for Supplementary Fig. 15c

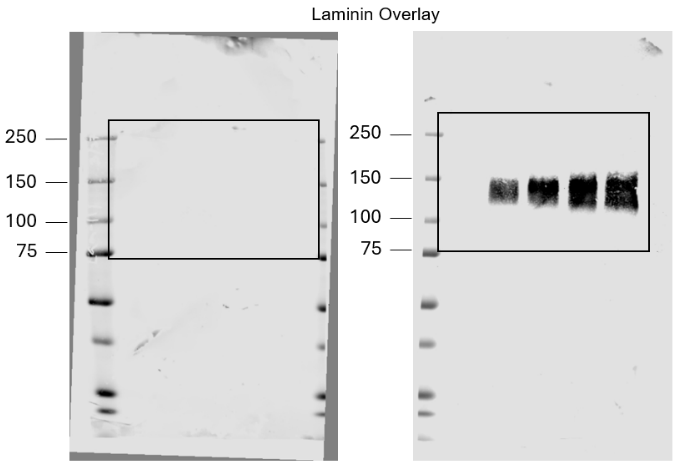

Supplement: Supplementary file 1 — Supplementary Information [file 41467_2025_64080_MOESM1_ESM.pdf]
