# Supplementary material for: LARGE1 processively polymerizes length-controlled matriglycan on prodystroglycan
Source: Nat Commun. 2025 Oct 10;16:9028. doi: 10.1038/s41467-025-64080-z (PMC12514197; doi:10.1038/s41467-025-64080-z)
Supplement: Supplementary file 2 — Reporting Summary [file 41467_2025_64080_MOESM2_ESM.pdf]

nature portfolio

Corresponding author(s): Kevin Campbell

Last updated by author(s): Aug 22, 2025

Reporting Summary

Nature Portfolio wishes to improve the reproducibility of the work that we publish. This form provides structure for consistency and transparency in reporting. For further information on Nature Portfolio policies, see our Editorial Policies and the Editorial Policy Checklist.

Statistics

For all statistical analyses, confirm that the following items are present in the figure legend, table legend, main text, or Methods section.

n/a	Confirmed	
	☒	The exact sample size (*n*) for each experimental group/condition, given as a discrete number and unit of measurement
	☒	A statement on whether measurements were taken from distinct samples or whether the same sample was measured repeatedly
☒		The statistical test(s) used AND whether they are one- or two-sided<br>*Only common tests should be described solely by name; describe more complex techniques in the Methods section.*
☒		A description of all covariates tested
☒		A description of any assumptions or corrections, such as tests of normality and adjustment for multiple comparisons
	☒	A full description of the statistical parameters including central tendency (e.g. means) or other basic estimates (e.g. regression coefficient) AND variation (e.g. standard deviation) or associated estimates of uncertainty (e.g. confidence intervals)
☒		For null hypothesis testing, the test statistic (e.g. *F*, *t*, *r*) with confidence intervals, effect sizes, degrees of freedom and *P* value noted<br>*Give P values as exact values whenever suitable.*
☒		For Bayesian analysis, information on the choice of priors and Markov chain Monte Carlo settings
☒		For hierarchical and complex designs, identification of the appropriate level for tests and full reporting of outcomes
☒		Estimates of effect sizes (e.g. Cohen's *d*, Pearson's *r*), indicating how they were calculated

Our web collection on statistics for biologists contains articles on many of the points above.

Software and code

Policy information about availability of computer code

Data collection	Titan Krios G3 with K3 direct electron detector (Gatan), SerialEM, Nanotemper NT.115, Li-COR Odessey CLx or M, Agilent 1260 Infinity HPLC with DAWN Helios II & Optilab T-rEX (Wyatt|Waters), Eiger2 XE 9M detector (Dectris), Bruker ultrafleXtreme MALDI-TOF/TOF, Shimadzu Scientific Prominence I, Nanodrop ND-1000 version 3.8.1
Data analysis	Relion (4.0.1), CryoSPARC (versions 3-4.5), Phenix 1.21.1, COOT 0.9.8.92, Pymol (2.4.0-3.0.4), Chimera 1.17.2, GraphPad Prism (9-10), Nanotemper MO.control (x86), ASTRA 8, BioXTAS RAW 2.1.1 & ATSAS 3.0.3, LabSolutions, Caver Analyst 2.0, Consurf, AlphaFold 3

For manuscripts utilizing custom algorithms or software that are central to the research but not yet described in published literature, software must be made available to editors and reviewers. We strongly encourage code deposition in a community repository (e.g. GitHub). See the Nature Portfolio guidelines for submitting code & software for further information.

Data

Policy information about availability of data

All manuscripts must include a data availability statement. This statement should provide the following information, where applicable:

- Accession codes, unique identifiers, or web links for publicly available datasets
- A description of any restrictions on data availability
- For clinical datasets or third party data, please ensure that the statement adheres to our policy

LARGE1 coordinates and maps have been deposited with the accession codes 7UI6 [https://www.rcsb.org/structure/7UI6] and EMD-26540 (C1 symmetry: [https://www.ebi.ac.uk/emdb/EMD-26540]) and 7UI7 [https://www.rcsb.org/structure/7UI7] and EMD-26541 (C2 symmetry: [https://www.ebi.ac.uk/emdb/EMD-26541]).

The map and model for LARGE1dTM with DGN are also deposited in the PDB under the accession code 9E1T [https://www.rcsb.org/structure/9E1T] and EMD-47420 [https://www.ebi.ac.uk/emdb/EMD-47420]. Glycoproteomic mass spectrometric data for LARGE1dTM have been deposited in PRIDE database under accession code PXD060053 [https://www.ebi.ac.uk/pride/archive/projects/PXD060053]. SAXS data have been deposited in the SASDB repository [https://www.sasbdb.org/data/SASDNF8/], [https://www.sasbdb.org/data/SASDNG8/], [https://www.sasbdb.org/data/SASDNH8/], [https://www.sasbdb.org/data/SASDNJ8/].

Research involving human participants, their data, or biological material

Policy information about studies with human participants or human data. See also policy information about sex, gender (identity/presentation), and sexual orientation and race, ethnicity and racism.

Reporting on sex and gender	N/A
Reporting on race, ethnicity, or other socially relevant groupings	N/A
Population characteristics	N/A
Recruitment	N/A
Ethics oversight	N/A

Note that full information on the approval of the study protocol must also be provided in the manuscript.

Field-specific reporting

Please select the one below that is the best fit for your research. If you are not sure, read the appropriate sections before making your selection.

☒ Life sciences ☐ Behavioural & social sciences ☐ Ecological, evolutionary & environmental sciences

For a reference copy of the document with all sections, see nature.com/documents/nr-reporting-summary-flat.pdf

Life sciences study design

All studies must disclose on these points even when the disclosure is negative.

Sample size	The size of light scattering and cryo-EM sample were limited to the amount of protein and time on the instrument but was sufficient for reconstructions using sets of final particles.
Data exclusions	Excluded particles classes of low resolution in cryo-EM reconstructions
Replication	Microscale thermophoresis were performed in triplicate. SEC-MALS-SAXS on at least three preparations and yielded similar parameters and molecular envelopes. LARGE1dTM enzymatic activity was performed using three different constructs of prodystroglycan 28-749, 28-398 and 28-340 and produced similar results.
Randomization	Not applicable for experiments used in this study.
Blinding	For all Western blotting experiments using mouse tissues, there was a positive or negative control, and all results were obtained in parallel under the same experimental conditions.

Reporting for specific materials, systems and methods

We require information from authors about some types of materials, experimental systems and methods used in many studies. Here, indicate whether each material, system or method listed is relevant to your study. If you are not sure if a list item applies to your research, read the appropriate section before selecting a response.

Materials & experimental systems

n/a	Involved in the study	
☐	☒	Antibodies
☐	☒	Eukaryotic cell lines
☒	☐	Palaeontology and archaeology
☐	☒	Animals and other organisms
☒	☐	Clinical data
☒	☐	Dual use research of concern
☒	☐	Plants

Methods

n/a	Involved in the study	
☒	☐	ChIP-seq
☒	☐	Flow cytometry
☒	☐	MRI-based neuroimaging

Antibodies

Antibodies used	Anti-matriglycan (IIH6), anti-laminin antibody, anti-dystroglycan N-terminal domain (1D9), anti-dystroglycan (AF6868)
Validation	All antibodies used have been previously published:<br>Anti-matriglycan (IIH6) mouse IgM (Campbell laboratory/Developmental Studies Hybridoma Bank, Hybridoma product IIH6 C4, AB_2617216) RRID:AB_2617216<br>Anti-mouse IgM; goat polyclonal, Li-COR biosciences Cat# 926–32280, RRID:AB_2814919<br>laminin mouse protein natural (ThermoFisher Scientific) Catalog #: 23017015<br>anti-laminin antibody produced in rabbit (Millipore-Sigma L9393, RRID:AB_477163)<br>anti-dystroglycan N-terminal domain (1D9) monoclonal mouse IgG<br>anti-dystroglycan polyclonal sheep IgG Catalogue #: AF6868 (R&D Systems) RRID:AB_10891298<br>Anti-mouse IgG (H+L); donkey polyclonal, Li-COR Biosciences Cat# 926–32212, RRID:AB_621847<br>Anti-rabbit IgG (H+L); donkey polyclonal, Li-COR Biosciences Cat# 926–32213, RRID:AB_621848

Eukaryotic cell lines

Policy information about cell lines and Sex and Gender in Research

Cell line source(s)	HEK 293 Freestyle cell line from ThermoFisher Scientific, wildtype reference clone and LARGE1 KO Hap1 from Haplogen/ Horizon Discovery, wildtype and POMK KO HEK 293T is from Abcam (ab267313).
Authentication	Generation and characterization of Hap1 mutant cell lines: Hap1 cells (RRID: CVCL_Y019) are a haploid human cell line with an adherent, fibroblast-like morphology, originally derived from parent cell line KBM-7 (RRID: CVCL_A426). Wild-type C631 (a diploid cell line containing duplicated chromosomes of Hap1) have been purchased from Haplogen/Horizon Discovery and gene-specific knockout Hap1 cells have been generated by Haplogen/Horizon Discovery. LARGE1 HAP1 cell KO lines are tested by rescue by transducing adenovirally encoded WT LARGE1. Human POMK knockout HEK293T cell line (ab267313) has been selected using puromycin.
Mycoplasma contamination	Mycoplasma testing of control (C631) on 9/24/2020 and LARGE1 KO on 9/24/2020 Hap1 cells as well as HEK 293 Freestyle parental line on 10/19/2020, and those expressing LARGE1dTM on 10/2/2020 and prodystroglycan on 10/2/2020 were performed to ensure the cells are not contaminated. Cell lines HEK 293T POMK KO and wildtype have been tested for mycoplasma by Abcam and are confirmed to be negative.
Commonly misidentified lines<br>(See ICLAC register)	The identity of Hap1 cells has been authenticated by the company using the STR profiling method.

Animals and other research organisms

Policy information about studies involving animals; ARRIVE guidelines recommended for reporting animal research, and Sex and Gender in Research

Laboratory animals	C57BL/6J wildtype and dystroglycan T190M knock-in mice were originally generated by Hara et al 2011 NEJM
Wild animals	This study did not involve wild animals.
Reporting on sex	The sex of mice was not considered in this study.
Field-collected samples	No field collected samples were used in this study.
Ethics oversight	Animal care, ethical usage, and procedures were performed in strict accordance with protocols approved by the National Institutes of Health and the Institutional Animal Care Use and Committee (IACUC), University of Iowa (#3051122). Mice were socially housed (unless single housing was required), under specific-pathogen-free conditions in an Association for Assessment and Accreditation of Laboratory Animal Care (AAALAC)-accredited animal facility. Mouse housing conditions were as specified in the Guide for the Care and Use of Laboratory Animals (National Research Council).

Note that full information on the approval of the study protocol must also be provided in the manuscript.

Plants

Seed stocks	N/A
Novel plant genotypes	N/A
Authentication	N/A
