# Supplementary figures and images for: LARGE1 processively polymerizes length-controlled matriglycan on prodystroglycan
Source: Nat Commun. 2025 Oct 10;16:9028. doi: 10.1038/s41467-025-64080-z (PMC12514197; doi:10.1038/s41467-025-64080-z)

1d

Coomassie stained

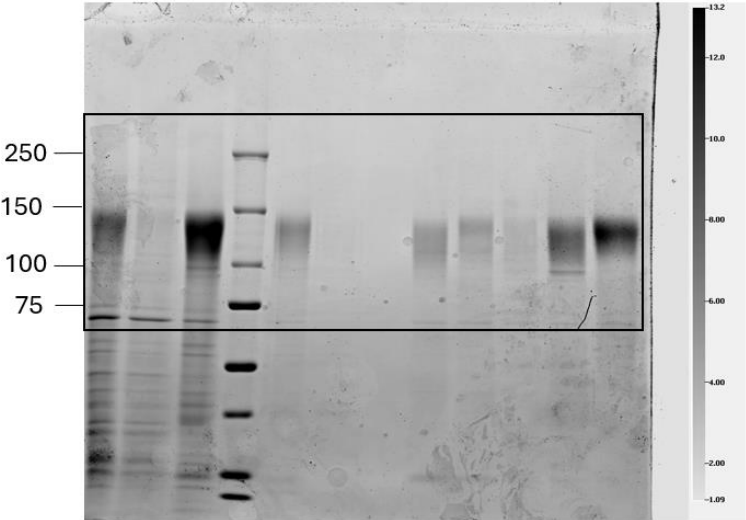

Matriglycan (IIH6)

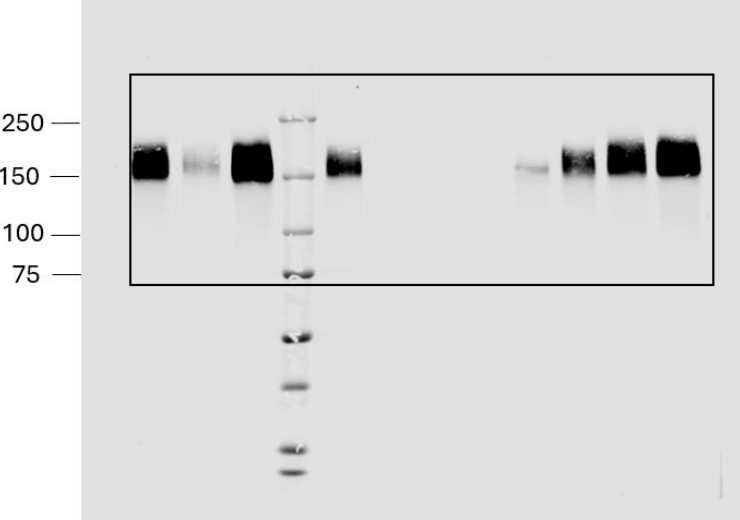

Dystroglycan N-terminal (1D9)

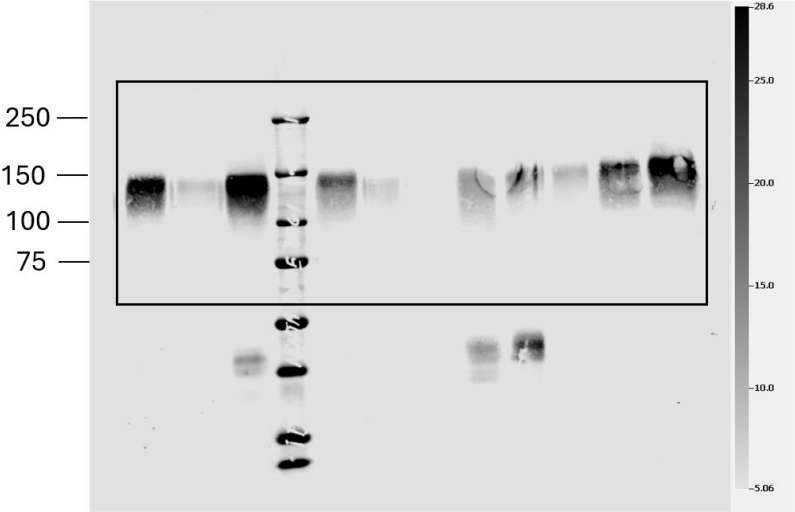

Laminin Overlay

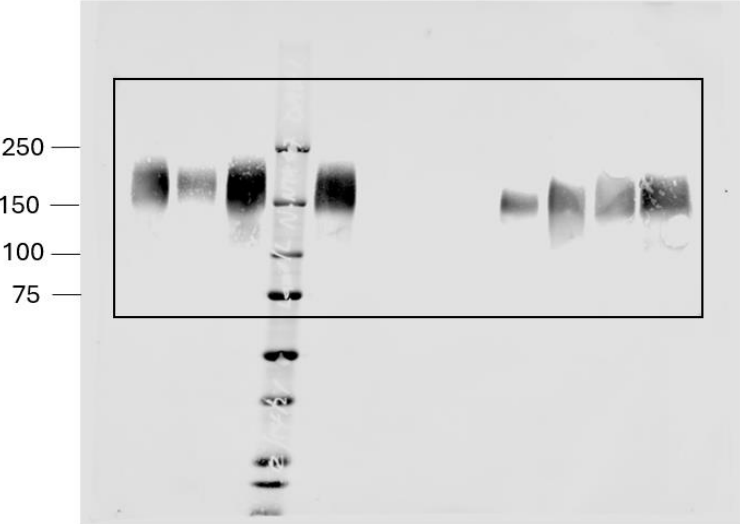

Supplement: Supplementary file 4 — Source Data [file 41467_2025_64080_MOESM4_ESM.zip › Data Files/Figure 1d - Full Figure.pdf]

1e

Matriglycan (IIH6)

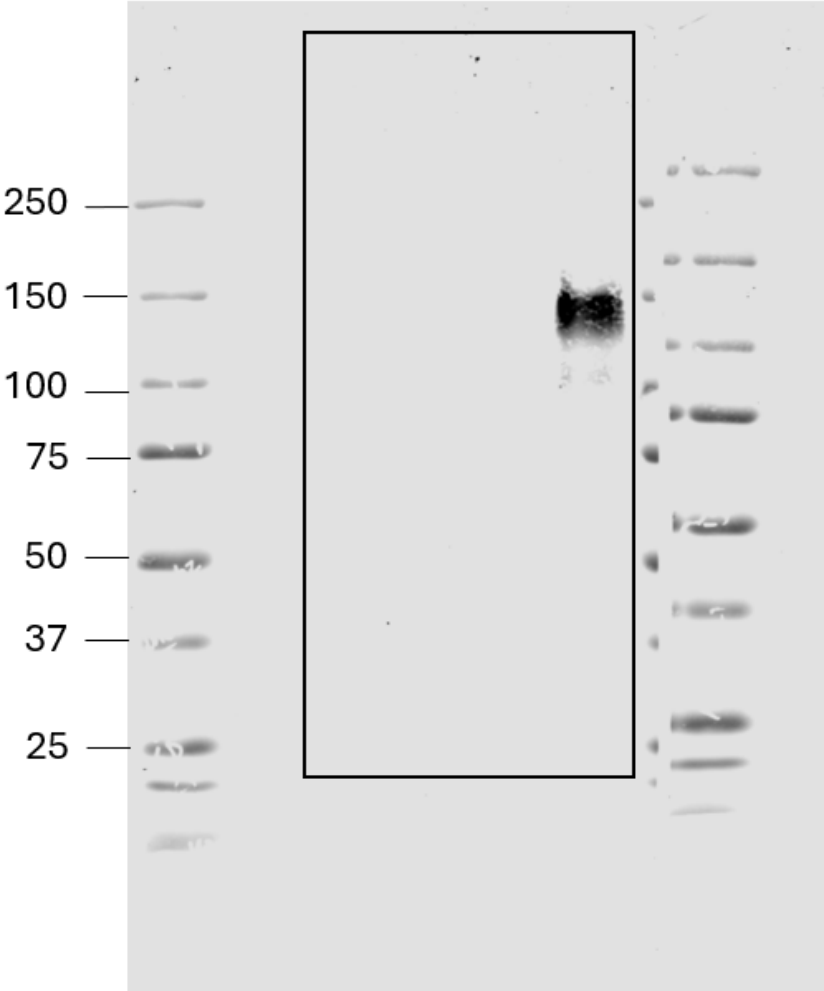

Laminin Overlay

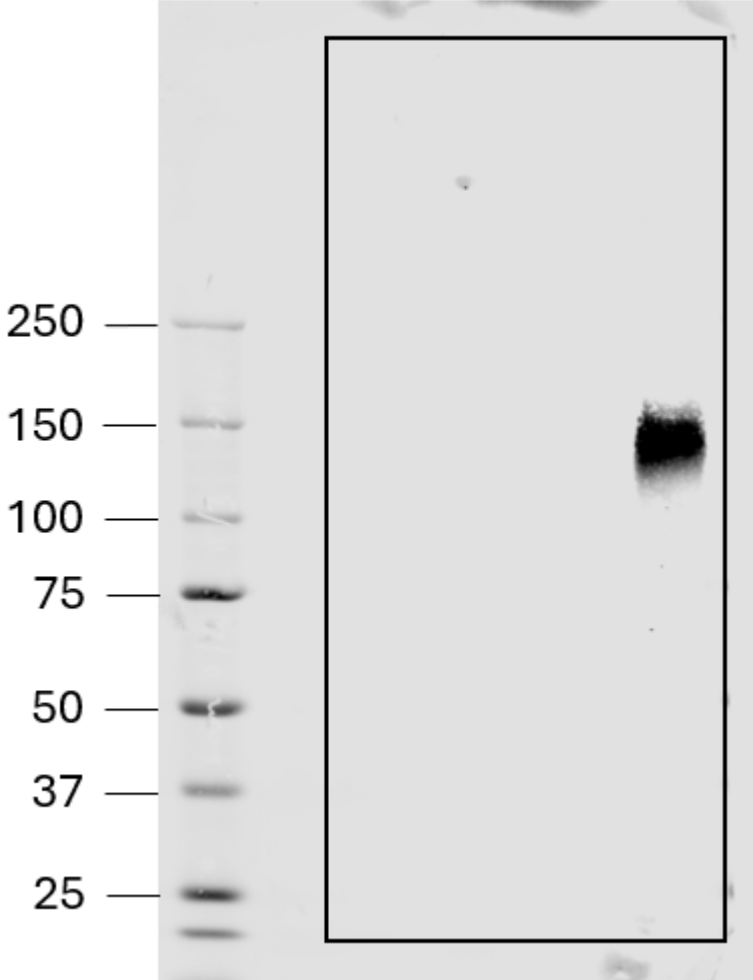

Core Dystroglycan (AF6868)

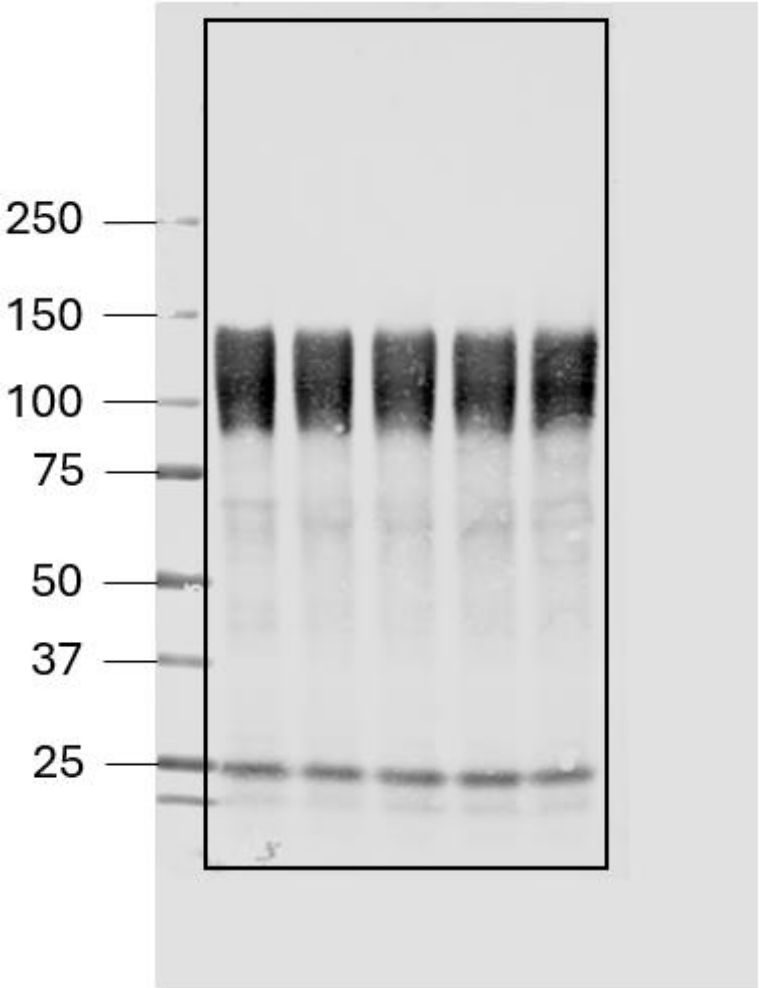

Supplement: Supplementary file 4 — Source Data [file 41467_2025_64080_MOESM4_ESM.zip › Data Files/Figure 1e - Full Figure.pdf]

3b

Matriglycan (IIH6)

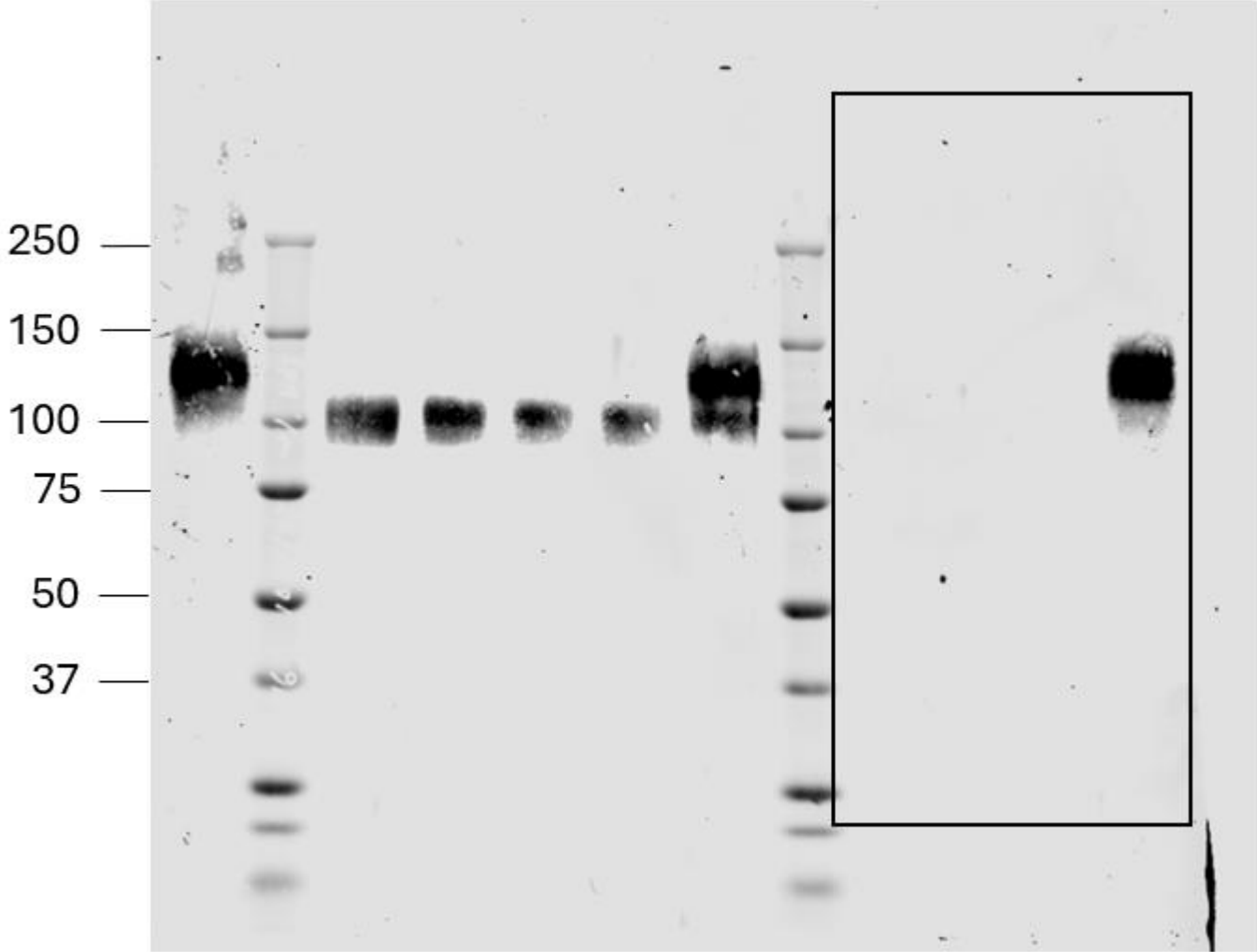

Laminin Overlay

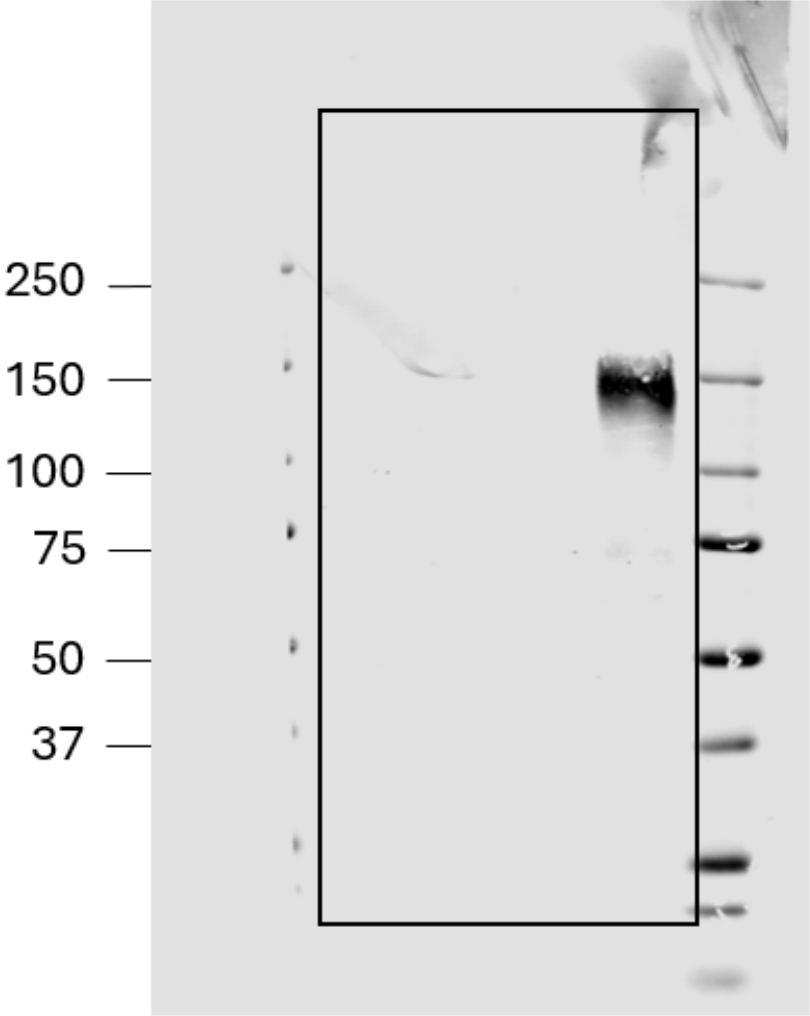

Supplement: Supplementary file 4 — Source Data [file 41467_2025_64080_MOESM4_ESM.zip › Data Files/Figure 3b - Full Figure.pdf]

3d

Matriglycan (IIH6)

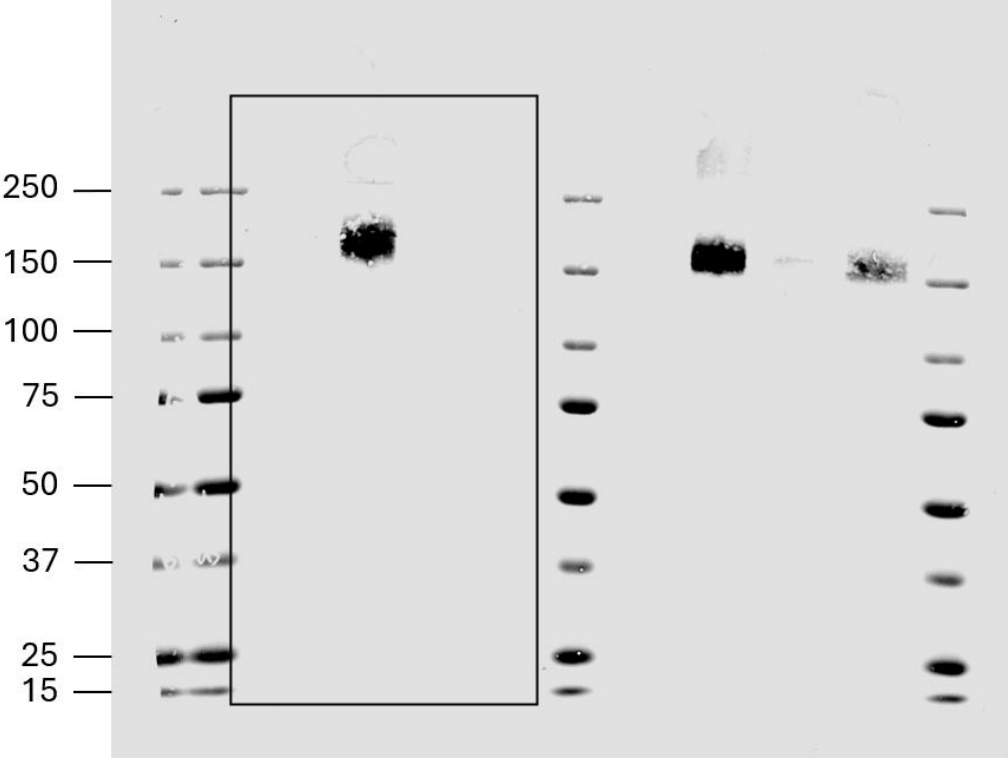

Core Dystroglycan (AF6868)

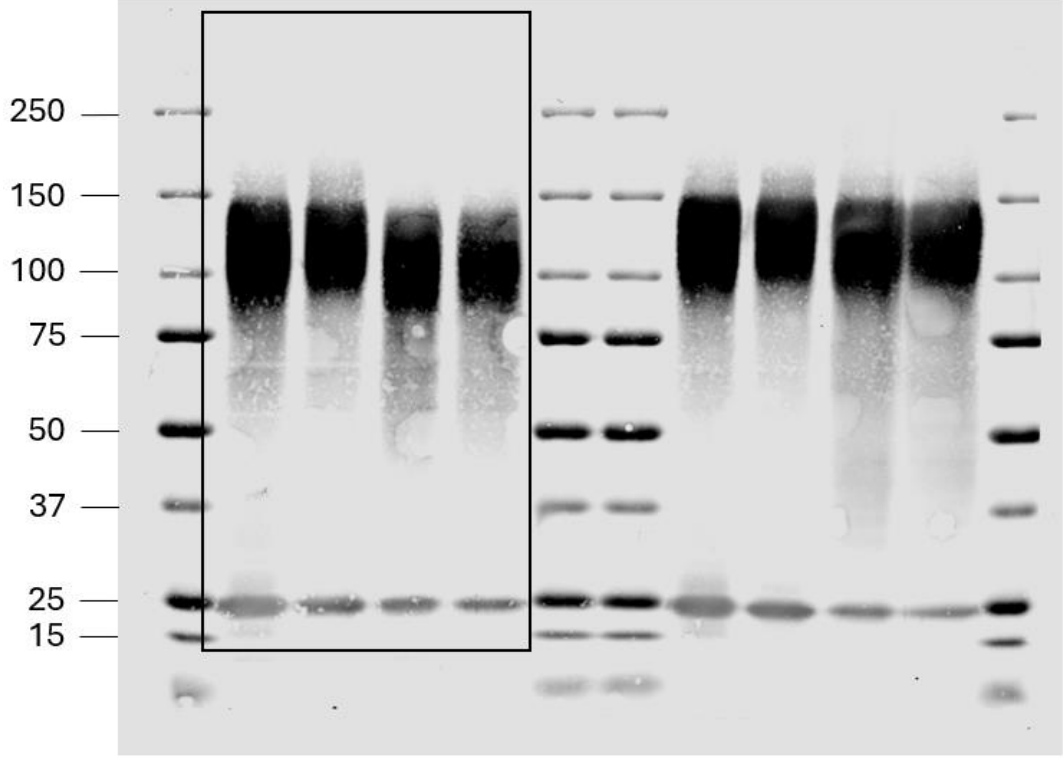

Supplement: Supplementary file 4 — Source Data [file 41467_2025_64080_MOESM4_ESM.zip › Data Files/Figure 3d - Full Figure.pdf]

Matriglycan (IIH6)

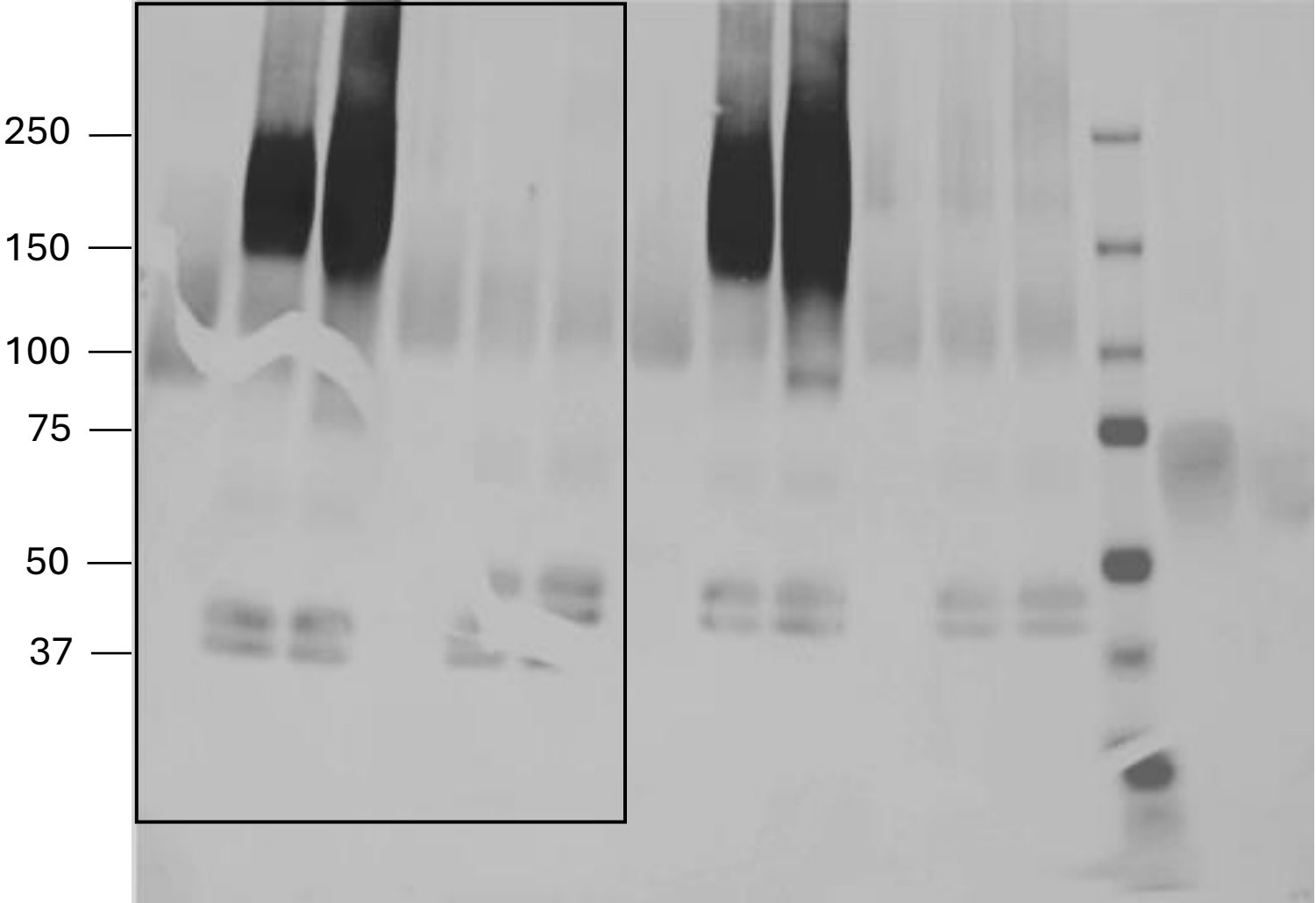

Supplement: Supplementary file 4 — Source Data [file 41467_2025_64080_MOESM4_ESM.zip › Data Files/Figure 3e - Full Figure.pdf]

4b

Matriglycan (IIH6)

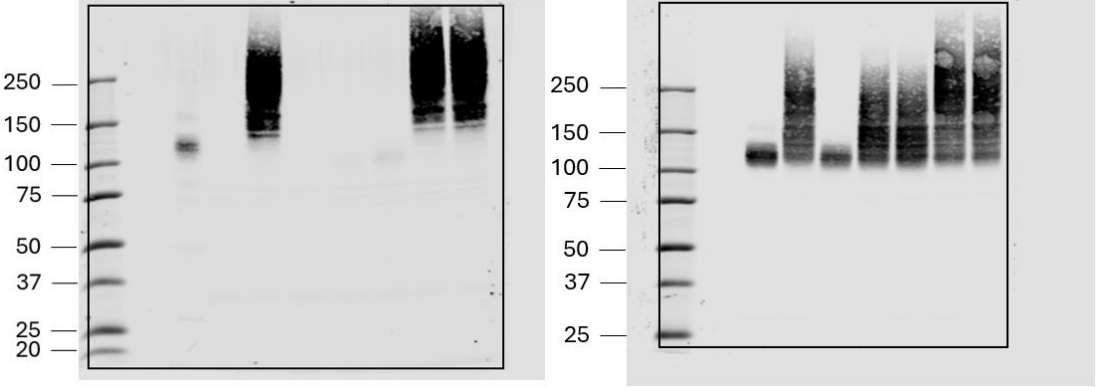

Laminin Overlay

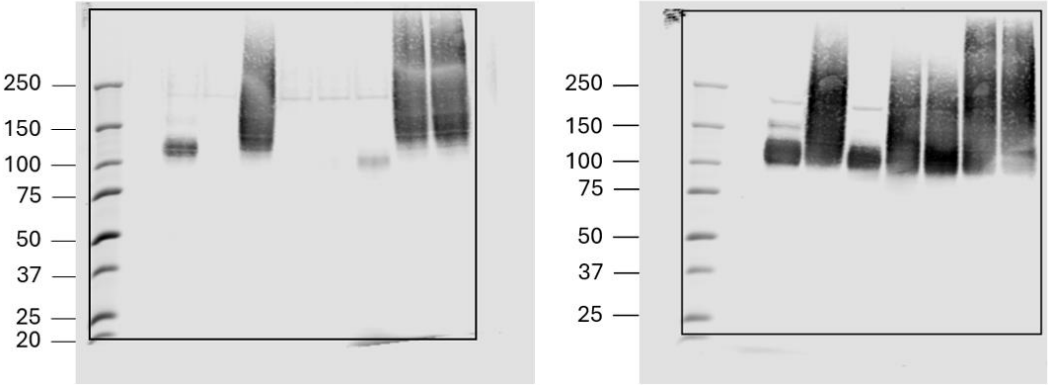

Core Dystroglycan (AF6868)

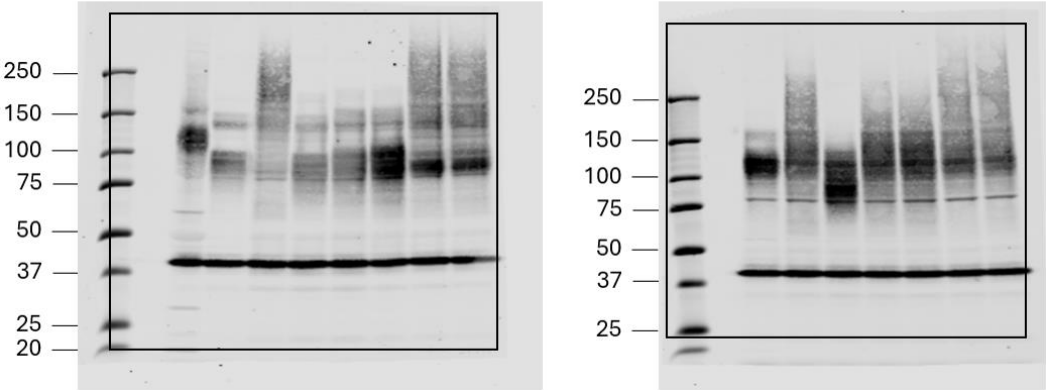

Supplement: Supplementary file 4 — Source Data [file 41467_2025_64080_MOESM4_ESM.zip › Data Files/Figure 4b - Full Figure.pdf]

4d

Matriglycan (IIH6)/DGN (1D9)

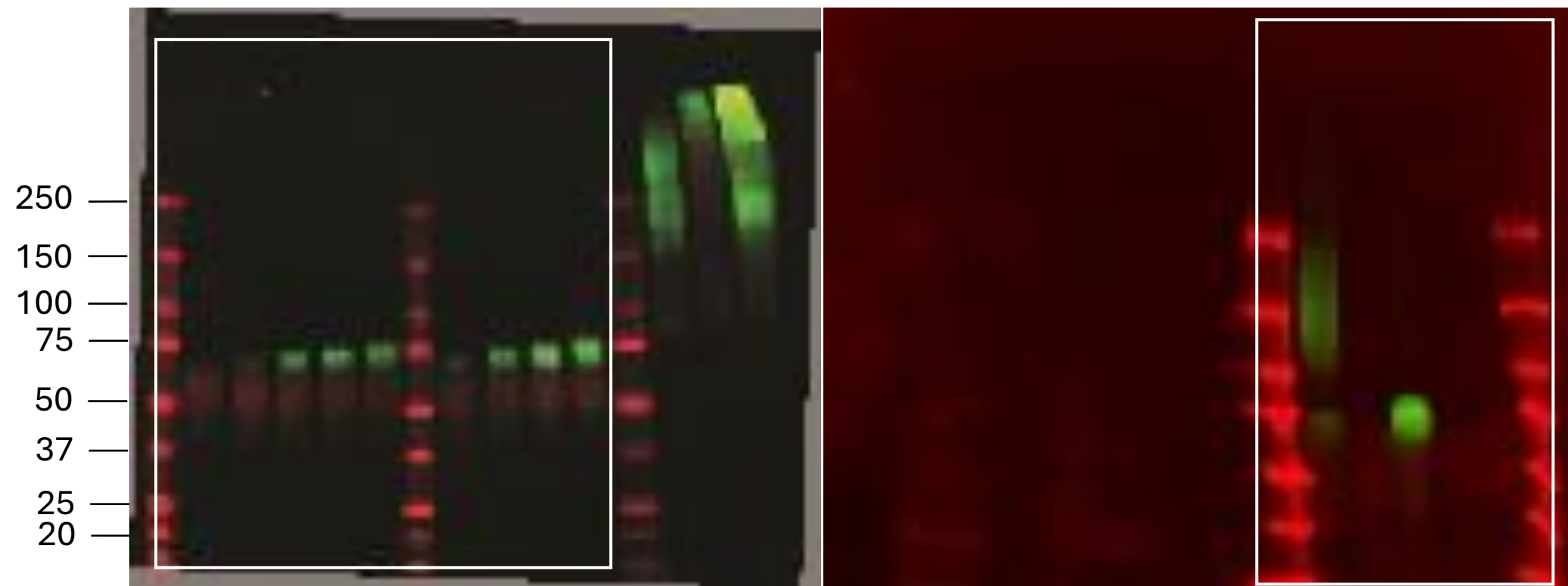

Supplement: Supplementary file 4 — Source Data [file 41467_2025_64080_MOESM4_ESM.zip › Data Files/Figure 4d - Full Figure.pdf]

5d

Matriglycan (IIH6)

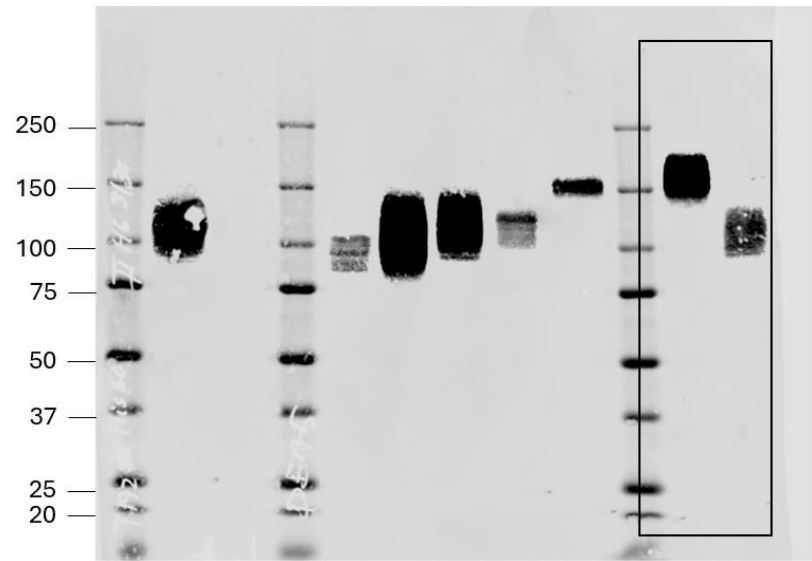

Core Dystroglycan (AF6868)

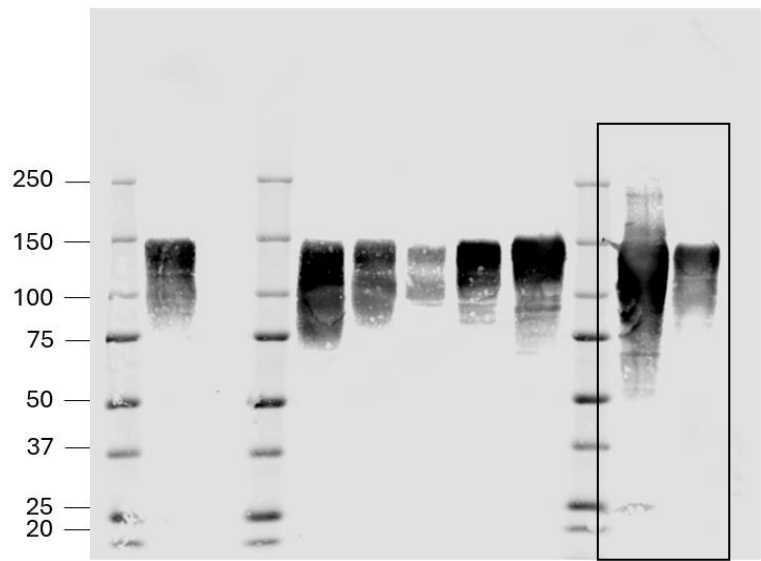

Coomassie Blue

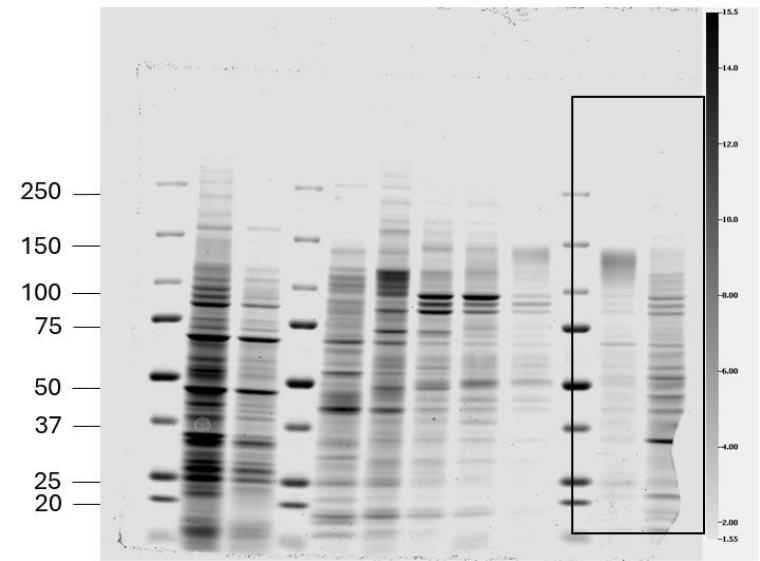

Supplement: Supplementary file 4 — Source Data [file 41467_2025_64080_MOESM4_ESM.zip › Data Files/Figure 5d - Full Figure.pdf]

Laminin Overlay

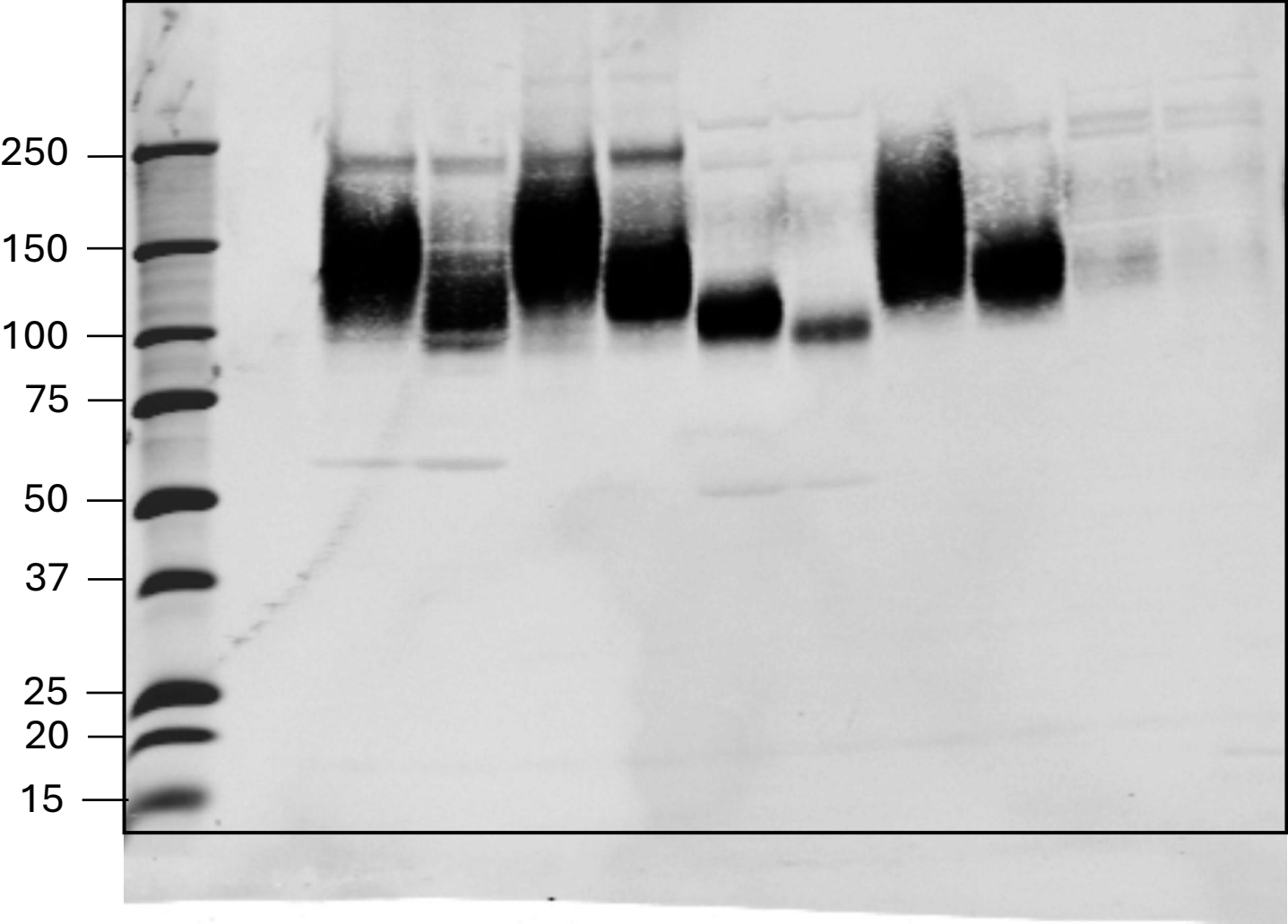

Supplement: Supplementary file 4 — Source Data [file 41467_2025_64080_MOESM4_ESM.zip › Data Files/Figure 5e - Full Figure.pdf]
